# Hepatitis B Virus Infection Among Leprosy Patients: A Case for Polymorphisms Compromising Activation of the Lectin Pathway and Complement Receptors

**DOI:** 10.3389/fimmu.2020.574457

**Published:** 2021-02-11

**Authors:** Angelica Beate Winter Boldt, Camila de Freitas Oliveira-Toré, Gabriela Canalli Kretzschmar, Hellen Weinschutz Mendes, Sérvio Túlio Stinghen, Fabiana Antunes Andrade, Valéria Bumiller-Bini, Letícia Boslooper Gonçalves, Anna Carolina de Moraes Braga, Ewalda von Rosen Seeling Stahlke, Thirumalaisamy P. Velavan, Steffen Thiel, Iara José Taborda de Messias-Reason

**Affiliations:** ^1^ Laboratory of Human Molecular Genetics, Postgraduate Program in Genetics, Department of Genetics, Federal University of Paraná, Curitiba, Brazil; ^2^ Laboratory of Molecular Immunopathology, Postgraduate Program in Internal Medicine and Health Sciences, Department of Clinical Pathology, Hospital de Clínicas, Federal University of Paraná, Curitiba, Brazil; ^3^ Health State Department of Paraná, Curitiba, Brazil; ^4^ Institute of Tropical Medicine, Universitätsklinikum Tübingen, Tübingen, Germany; ^5^ Vietnamese-German Center for Medical Research, Hanoi, Vietnam; ^6^ Faculty of Medicine, Duy Tan University, Da Nang, Vietnam; ^7^ Department of Biomedicine, Aarhus University, Aarhus, Denmark

**Keywords:** mannose-binding protein-associated serine proteases, mannose-binding lectin, ficolin, leprosy, complement 3b receptors, genetic polymorphisms, complement system proteins, Hepatitis B

## Abstract

Thousands of leprosy patients not only suffer from physical deformities, but also either have or have had hepatitis B virus (HBV) coinfection. Polymorphisms of the complement system modulate susceptibility to leprosy, but genetic susceptibility to past or present HBV infection is unknown. We used sequencing and multiplex sequence-specific PCR to genotype 72 polymorphisms of seven genes (*MBL2*, *FCN1, FCN2, FCN3, MASP1, MASP2, C3*) encoding components of the lectin pathway, and two genes encoding complement receptors (*CR1, VSIG4*) in 190 patients, of which 74 were positive for HBsAg and/or anti-HBc (HBV+, 93.2% with a resolved infection) and 116 lepromatous patients, and 408 HBV-blood donors. In addition, we tested for levels of proteins of the lectin pathway. We found no difference between serum concentrations of mannan-binding lectin (MBL), MBL-associated serine proteins (MASP-1, MASP-2, MASP-3, MAp44), ficolin-3 (FCN-3), soluble complement receptor 1 (sCR1) and MBL mediated C4 activation, measured by ELISA or TRIFMA in up to 167 HBV+ and HBV− patients. Haplotypes lowering protein levels or encoding dysfunctional proteins increased susceptibility to HBV infection: MBL2*LYQC (OR = 3.4, p = 0.02), MASP1*AC_CC (OR = 4.0, p = 0.015) and MASP2*1C2-l (OR = 5.4, p = 0.03). Conversely, FCN1*3C2 haplotype, associated with higher gene expression, was protective (OR = 0.56, P = 0.033). Other haplotypes associated with HBV susceptibility were: MASP2*2B1-i (OR = 19.25, P = 0.003), CR1*3A (OR = 2.65, P = 0.011) and VSIG4*TGGRCG (OR = 12.55, P = 0.014). Some polymorphisms in ficolin genes associated with lower protein levels increased susceptibility to leprosy/HBV infection: FCN*1 (OR = 1.66, P = 0.029), FCN2*GGGCAC (OR = 6.73, P = 0.008), and FCN3*del_del_C (OR = 12.54, P = 0.037), and to lepromatous disease/HBV infection: FCN2*TA (OR = 2.5, P = 0.009), whereas FCN2*MAG was associated with increased FCN-2 expression and resistance against coinfection (OR = 0.29, P = 0.026). These associations were independent of demographic factors and did not increase susceptibility to leprosy *per se*, except MASP2*1C2-l. Associations for *FCN2, FCN3, MASP1, MASP2*, and *VSIG4* variants were also independent of each other. In conclusion, polymorphisms compromising activation of the lectin pathway of complement increase susceptibility to HBV infection, with ficolin polymorphisms playing a major role in modulating the susceptibility among leprosy patients.

## Introduction

Leprosy is an ancient disease that has plagued humanity through the ages. Although currently under control, it is still a big health problem in underdeveloped countries (1). Brazil’s leprosy detection rate ranged from 10.9 to 78.4 per 100,000 inhabitants from 2001 to 2016, the second-highest rate worldwide. It is hyperendemic among children under 15 years of age, leaving a trail of irreversible disabilities behind, especially in boys (2).

Part of the susceptibility to leprosy and its broad spectrum of symptoms is genetically determined. Individuals with a strong cellular immune response can overcome the disease without even noticing the infection, whereas those who rely on a humoral response acquire a widespread disease that ultimately leads to blindness and gross deformities if left untreated (3). This is the cause of social stigmatization, which historically encouraged the isolation of affected individuals in “institutions” and “colonies”. Although most of these institutions have been inactivated in Brazil, especially with the introduction of multidrug therapy (MTD), important remnants still present a high prevalence. They are historically recognized as former leprosy colonies, *e.g.*, the Prata Village, where MTD therapy resistance recently emerged (4).

Early exposure, late detection, and constant transmission of *Mycobacterium leprae* increase the probability of coinfections. In addition, confinement to a limited space is known to increase the spread of other infections (5–9). Furthermore, the same immune and genetic components that modulate the susceptibility to leprosy may modulate the susceptibility to coinfections, as well. This prompted our former epidemiological investigation on coinfections in South-Brazilian leprosy patients. Among those investigated, past infection with hepatitis B virus (HBV) emerged as the most frequent outcome (60%) compared to human immunodeficiency virus (HIV-1) (0.5%), hepatitis C virus (HCV) (3.5%), Chagas disease (4.5%), HTLV (0), and syphilis (9%) (5, 10). In fact, the frequency of HBV infection among leprosy patients was ten times higher than among the general Brazilian population (6%). In that study, it became clear that both diseases are strongly associated, and that institutionalization poses leprosy-affected individuals to a higher risk for HBV infection (10).

WHO estimates that 257 million individuals worldwide live with HBV (11). The worldwide prevalence of chronic HBV infection has been estimated at 3.9% (12), from about 0.7–1.6% in the Americas and Europe to 6.1–6.2% in African and Western Pacific regions, respectively. However, it reaches disproportionally high values in some Southeast Asia countries, as 10.8% in Vietnam (13). Among 50–80% of those chronically infected develop hepatocellular carcinoma, and 70–80% of these cases occur in patients with cirrhosis (14). Innate immunity factors that predispose to HBV infection and chronic complications are also involved in the susceptibility to leprosy and lepromatous disease, *e.g.*, polymorphisms of killer-cell immunoglobulin-like receptors (*KIR*) and human major histocompatibility complex class I chain-related gene A (*MICA*) genes (15–18).

Among innate immunological factors that drive susceptibility to infection and disease progression, the lectin pathway of complement (LP) occupies a prominent position. The LP starts typically with the recognition of sugar moieties or acetylated residues on pathogens or altered/damaged cells by the pattern recognition molecules (PRMs) such as the collectin mannan-binding lectin (MBL) or ficolins (FCNs), respectively. These pattern-recognition molecules are associated with homodimers of serine proteases (MASPs). When such PRM/MASP complexes bind to a surface, MASP-1 may autoactivate and then activates MASP-2. MASP-2 further cleaves complement factor C4, and MASP-1 and MASP-2 cleave C2 complement components, building up the complex of C4bC2a, which is a C3 convertase. This molecule cleaves C3 and channels the cascade further to cover C3b on the target, causing its opsonization, phagocyte internalization, and destruction. This step is a major outcome of complement activation, common to all three initiation pathways (including the classical and alternative pathways). The final steps of the proteolytic cascade lead to the assembly of the C5 convertase (C4bC2aC3b), subsequent cleavage of C5 and C5b aggregation of C6–C7 components, which pierce membrane-attack complexes through the addition of C8 and some of 10–18 C9 subunits on the target’s surface [reviewed by (19, 20)].

The LP has long been recognized as playing a critical role in defense against various pathogens and has recently been suggested as a target for controlling SARS-CoV-2 infections (21). Viral infections may spread opportunistically among already immunocompromised patients, regardless of their cause. Within this scenario, the LP’s inflammation generating functions may act as a “double-edged sword”—low levels of its components may increase the chance for coinfections, whereas the opposite may accelerate tissue damage and aggravate the disease course. Leprosy poses a paradox to this problem since *M. leprae* relies on opsonization, one of the major outcomes of complement activation, to enter the host cell. Thus, it seems to usurp the LP to succeed, and deficiency/low levels of LP components have been associated with disease protection. The polarization of the disease in a relatively small proportion of individuals that do not get spontaneously cured places another layer of complexity to this immunopathological problem. The same genetic variants associated with resistance against leprosy may increase the risk to a lepromatous, Th2-associated clinical presentation or foster adverse treatment responses, as reversal (type 1) reactions and erythema nodosum leprosum (type 2) reactions (reviewed by 19).

Furthermore, individuals at higher risk for leprosy often share a low socio-economic background and hygiene environment that foster other infections. Past/present HBV infections in leprosy patients are not uncommon, reaching figures between 26 and 60% in Brazil (9, 10). However, as opposed to leprosy, a highly efficient immunological response associated with the complement cascade is necessary to abort infection. Notwithstanding, the same cascade may, as in the case of leprosy, cause more harm than good in chronic HBV infection and influence the clinical course towards adverse outcomes (22). This study enabled us to identify different, sometimes opposite associations comparing healthy, HBV-negative individuals and leprosy-affected, HBV-infected, and uninfected patients. This pioneering approach also brought new insights into the LP role and complement receptors on HBV infection and unprecedented findings on the genetic susceptibility to HBV/leprosy coinfection.

## Materials and Methods

### Ethics Statement

All participants in this study were adults (over 18 years of age) and signed written informed consent, previously approved by the local medical ethics committee of the HC-UFPR (protocol 497.079/2002–06, 218.104 and 279.970). We designed this case–control, cross-sectional study and conducted it according to the Declaration of Helsinki.

### Research Participants

We included 190 from a cohort of 199 leprosy patients who either attended the Federal University of Paraná’s Clinical Hospital (HC-UFPR), Paraná’s Hospital of Sanitary Dermatology (HDSPR) or the Regional Center of Specialities-Barão (CRS-Barão), as formerly described (10). We excluded nine individuals due to a lack of information for past/present HBV infection. All individuals were recruited in 2002–2003 when most of the Brazilian population was still not enrolled in the National Immunization Program for HBV vaccination. This program started in 1998 with a particular focus on newborn individuals (23). Of the 190 patients, 59 lived in the HDSPR at the recruitment time (31.1%).

According to the clinical classification proposed by Ridley and Jopling (24), 116 patients presented lepromatous leprosy (61.1%), 29 borderline leprosy (15.3%), 14 tuberculoid leprosy (7.4%), and 10 an undetermined form of leprosy (5.3%). Twenty-one (11.1%) were diagnosed with unspecified leprosy and excluded from any analysis comparing lepromatous and non-lepromatous patients.

After written informed consent, 7 ml of venous blood without anticoagulant was collected from each patient to test specific HBV antigens and antibodies in serum. Aliquots of 0.5–1 ml were used for each complement-quantifying assay. We also collected 4 ml of blood with anticoagulant ethylenediaminetetraacetic acid (EDTA) for DNA extraction from peripheral blood mononuclear cells through a commercial kit (Qiagen GmbH, Hilden, Germany), following the manufacturer’s instructions.

The samples were tested for past/present HBV infection, as formerly described (10). Briefly, we identified anti-HBc (antibodies against HBV core antigen) using ELISA (MONOLISA ^®^ a-HBc PLUS, BIO-RAD, Marnes La Coquette—France). This antibody is indicative of past HBV infection. We also searched for HBsAg, a marker of active HBV infection, with a sandwich ELISA (MONOLISA ^®^ a-HBc PLUS, BIO-RAD, Marnes La Coquette—France). If positive for anti-HBc alone (HBsAg negative), patients were tested by microparticle enzyme immunoassay to detect antibodies against hepatitis B surface antigen (anti-HBs, a marker also used for positive vaccination response) (Murex anti-HBs, Murex Biotech Limited, Temple Hill, UK). All positive samples were retested using the same methods. Only five of 74 HBV positive individuals (6.8%) presented active HBV infection (positivity for HBsAg). Of these, two presented acute and three, chronic HBV infection. All others were positive for anti-HBc, indicative of a past resolved HBV infection. None was positive for anti-HBs alone, and none presented HBV-related liver cirrhosis or hepatocellular carcinoma. Of the 59 HDSPR-institutionalized patients, 36 were HBV+ (61%).

For practical reasons, we considered leprosy patients with past/present HBV infection as “HBV positive” (HBV+), whereas those negative for the tested markers, as “HBV negative” (HBV−). The HBV+ patients were older than HBV− patients, but the distribution of sex and ethnic groups did not differ among the patients (**Table 1**). We also included different groups of HBV− blood donors as control groups, depending on the investigated gene, from HC-UFPR, Hemepar, and Hospital Evangélico blood banks (**Table 2**). Their age and sex distributions were described before (25–31).

**Table 1:** Characterization of leprosy patients according to past/present HBV infection.

	Patients		P value	Lepromatous		P value	Non-lepromatous		P value
	HBV−	HBV+		HBV−	HBV+		HBV−	HBV+	
N	116	74		63	53		40	13	
Female (%)	47 (40.5)	25 (33.8)	ns	22 (34.9)	16 (30.2)	ns	22 (55.0)	6 (46.2)	ns
Average age (min–max)	50.5 (19–82)	57.1 (24–94)	0.0003	48.0 (20–73)	59.9 (30–94)	<0.0001	48.5 (19–75)	47.9 (31-67)	ns
Afro-Brazilian (%)	18 (15.5)	19 (25.7)	ns	9 (14.3)	14 (26.4)	ns	8 (20.0)	4 (30.8)	ns

Age distributions were compared with Student’s t test, sex, and ethnic group distributions with Fisher’s exact test. ns, not significative.

**Table 2:** Sample sizes, genotyping method, and number of investigated polymorphism and haplotypes, per gene.

Genes	HBV− patients (ELISA/TRIFMA)	HBV+ patients (ELISA/TRIFMA)	Blood donors	Method	SNPs	Promoter	Coding (S/NS)	Intronic	UTR	eQTL	sQTL	Polymorph haplotypes(Total)	Reference
*MBL2*	116 (101, 30*)	74 (66, 16*)	200	Sanger Sequencing	19	14	4 (1/3)	0	1	3	0	8 (16)	(25)
*FCN1*	112	74	349	Multiplex SSP-PCR	7	6	0	0	1	5	1	7 (10)	(26)
*FCN2*	83	56	131	Sanger Sequencing	9	5	3 (1/2)	0	1	5	7	6 (8) 5 (5)	(27)
*FCN3*	92 (37)	51 (31)	146	Multiplex SSP-PCR	3	0	1 (frameshift)	2	0	3	2	3 (5)	(28)
*MASP1*	108 (79)	72 (60)	196	Multiplex SSP-PCR	5	0	0	2	3	4	1	10 (12)	(29)
*MASP2*	112 (22)	73 (24)	408	Multiplex SSP-PCR	11	1	6 (0/6)	3	1	9	8	9 (17)	(30)
*CR1*	96 (12)	56 (8)	198	Multiplex SSP-PCR	9	0	3 (0/3)	4	2	8	5	13 (15)	(31)
*VSIG4*	111	74	177	Multiplex SSP-PCR	6	2	1 (0/1)	3	0	4	0	4 (7)	—
*C3*	93 (49)^&^	60 (28)^&^	191	Multiplex SSP-PCR	3	0	2 (0/2)	1	0	3	0	4 (5)	—

SNP, single nucleotide polymorphism (S, synonymous; NS, non-synonymous); eQTL and sQTL, expression and splicing quantitative trait loci for the same or other gene, respectively (data from GTEx portal).

*MBL/C4 complexes (measure of LP activation).

^&^Evaluated with an immunoturbidimetric assay.

MBL2, mannan-binding lectin 2; FCN, ficolin; MASP, MBL-associated serine protease; CR1, complement receptor 1; VSIG4, V-set and immunoglobulin domain containing 4; C3, complement component 3.

SSP-PCR, sequence-specific polymerase chain reaction.

### Identification and Characterization of Genetic Variants

We focused on LP genes plus phagocytic complement receptors for two principal reasons: (1) the LP gene products do not primarily rely on antibodies and thus on the adaptive immune response to launch the complement cascade, as is the case for the classical pathway. It is thus the first to get activated after pathogen recognition, also playing an important role in activating the alternative pathway (through MASP-3); (2) PRMs of the LP readily recognize *Mycobacterium leprae* as well as HBV, mediating their phagocytosis through the investigated phagocytic receptors (which in the case of *M. leprae* may be beneficial for the parasite and establishment of infection). Furthermore, we chose SNPs whose alleles were: common in at least one of the major human ethnic groups and were either: (1) located in a regulatory region and associated with gene expression/protein levels, or (2) located in an exon and causing a structural/functional defect in the protein due to a missense or indel mutation.

We identified 72 single nucleotide polymorphisms (SNPs) of nine genes of the complement system (**Table 2**). Four genes that encode PRMs (*MBL2*—mannan-binding lectin, *FCN1*—M-ficolin, *FCN2*—L-ficolin and *FCN3*—H-ficolin), two that encode collectin and ficolin-associated serine proteases (*MASP1* and *MASP2 –* MBL-associated serine proteases), two that encode complement receptors (*CR1*—complement receptor 1 or CD35, *VSIG4*—V-set and immunoglobulin domain-containing 4 or CRIg), and one encodes the complement component C3 (*C3*). Among the selected 72 polymorphisms, 39% occur in the promoter region, 28% in exons (25% are non-synonymous substitutions), 21% in introns, and 13% in 5′ or 3′ untranslated regions (UTRs). Of all of these, 61.1% (44/72) are associated with mRNA levels and 33.3% (24/72) with alternative splicing of the pre-mRNA of the same gene or a neighboring gene (expression quantitative trait loci or eQTL and splicing quantitative trait loci or sQTL, respectively), according to data of the GTEx portal (https://gtexportal.org). These variants were distributed in 100 haplotypes, of which 69 are polymorph (with more than 1% frequency in blood donors) (**Table 2**).

We used Sanger sequencing to identify *MBL2* and *FCN2* genetic variants and multiplex sequence-specific PCR (SSP-PCR) to identify *FCN1, FCN3, MASP1, MASP2*, and *CR1* polymorphisms, as formerly described (25–31). Multiplex-SSP-PCR was also the choice technique for identifying C3 and *VSIG4* polymorphisms (primer sequences and PCR protocols are available in **Supplementary Table 1**).

### Measurement of Complement Proteins

MBL serum concentrations were measured in a double-antibody enzyme-linked immunosorbent assay (ELISA), with an in-house assay previously described by our group (32). Serum FCN-3 and MASP-2 concentrations and serum MBL-C4 activation complexes were measured as described before (19, 28, 30) with commercial ELISA kits (HK340, HK326, and HK327, respectively, from Hycult Biotechnology, Uden, The Netherlands). Serum sCR1 levels were measured as described before (31) by a commercial enzyme-linked immunosorbent assay (ELISA), using the SEB123Hu kit (USCN Life Science Inc., Wuhan, China), according to the manufacturer’s instructions. Color intensity was evaluated at 450 nm in an ELISA reader.

Serum MASP-1, MASP-3, and MAp44 levels were measured with time-resolved immunofluorimetric assays (TRIFMA), as published (29). In the MASP-3 and MAp44 TRIFMA, the bound protein is detected by a specific biotin-labeled monoclonal antibody, recognized by europium-labeled streptavidin. The provided signal is measured by time-resolved fluorometry (33). For measuring MASP-1, we used an inhibition assay. Circulating MASP-1 from the sample inhibits the binding of an anti-MASP-1 antibody to a surface coated with a fragment of MASP-1, followed by its detection, as previously described (34).

C3 serum levels were assessed using the automated immunoturbidimetric assay (Beckman Coulter, AU analyzer, USA) with reference values ranging from 87 to 200 mg/dl, the according to manufacturer’s protocol (Beckman Coulte, ref: OSR6159).

### Statistics

Genotype, allele, and haplotype frequencies were obtained by direct counting and compared using Fisher’s exact test and odds ratios with the respective 95% confidence limits. The expectation-maximization (EM) algorithm was used to reconstruct haplotypes not phased by SSP primers and calculate maximum likelihood estimates of haplotype frequencies while considering phase ambiguity. The EM calculations and the hypothesis of Hardy–Weinberg equilibrium test were performed with the ARLEQUIN software package version 3.1 (http://cmpg.unibe.ch/software/arlequin3/). We compared the distribution of protein serum concentrations between the groups, using non-parametric Mann–Whitney/Kruskal–Wallis tests (since their distribution did not pass the Shapiro–Wilk normality test), with Graphpad Prism 5.01 (GraphPad Software, La Jolla, CA).

The reduced multivariate logistic regression model was used to adjust results for demographic factors using STATA v.9.2 (StataCorp, TX, USA). The P values obtained with multiple association tests were corrected with the Benjamini–Hochberg method (35). We ended up with *cc*. 75% statistical power for most comparisons between common haplotypes with at least 20% frequency in HBV− leprosy patients and 10% in HBV+ patients (for uncommon haplotypes with 1 and 5% in each of these groups, respectively, it was 65%).

We used the following strategy for data interpretation: if the results comparing healthy controls with HBV+ individuals did not differ from the comparison between HBV+ and HBV− leprosy patients, we considered the association as independent of *M. leprae* infection and due to a higher susceptibility or resistance to HBV *per se*. This first analysis allowed us to disentangle HBV infection susceptibility from susceptibility to HBV/*M. leprae* coinfection. Results specific to the comparison within the leprosy group, between HBV+ and HBV− individuals, corresponded to increased susceptibility or resistance to both diseases. Finally, we also focused on possible associations within the lepromatous and non-lepromatous groups, which is particularly interesting due to their contrasting Th1 and Th2 immune responses, respectively.

Associated polymorphisms were further characterized according to data of the ENCODE (Encyclopedia of Noncoding Elements), available in the UCSC Genome Browser (https://genome.ucsc.edu/), Polyphen and SIFT scores from the Ensembl Genome Browser (https://www.ensembl.org/).

## Results

By comparing HBV- and leprosy- blood donors, we were able to identify gene associations due to susceptibility to HBV *per se* and differentiate them from genetic associations with susceptibility to leprosy only [already published in references (25–31)], and to both HBV and leprosy. The last may be interpreted as increased susceptibility to HBV infection in the already immunocompromised leprosy-patient group (coinfection) or higher HBV susceptibility of leprosy-prone individuals (HBV infection before *M. leprae* infection).

### Associations With Hepatitis B Virus Infection *Per Se*

We considered the following genetic associations as with HBV infection *per se* (independent from leprosy disease) if the direction of association did not differ between the comparisons of HBV+ leprosy patients and blood donors and the comparisons between HBV+ and HBV− leprosy patients, either at the level of haplotype frequencies (**Supplementary Tables 2**–**11**) and/or at the level of genotype frequencies (**Table 2**). Seven haplotypes of six different genes were associated with a dominant or an additive effect on the susceptibility or resistance to HBV infection after correcting for age and ethnic group distribution. We found no associations with protein serum levels of any tested complement component (**Supplementary Figure 1**) or tested *C3* polymorphisms (**Supplementary Table 11**).

Five frequent haplotypes were associated with increased susceptibility to HBV infection. Among them, as well as among all selected genes, the polymorphism of *MBL2* is particularly outstanding due to its common deficiency-causing variants. One of them is embedded in the *LYQC* haplotypes, associated with the disease in the multivariate logistic regression, independently of age and ethnic group distribution (OR = 3.38 [95%CI = 1.19–9.57], P = 0.022) (**Table 3**). Indeed, the summed frequencies of three different *LYQC* haplotypes identified by haplotype-specific sequencing did not differ between controls, HBV− leprosy, and HBV− lepromatous patients (*4F1-l, *4F2A-l with rs45602536 and *4F3-l with rs67990116 minor promoter alleles) (**Supplementary Table 2**). All *LYQC* haplotypes carry the minor allele of the rs1800451 polymorphism in codon 57, which disrupts the collagen Gly-Xaa-Yaa repeats by causing the substitution of glycine with glutamic acid (p.Gly57Glu, called the “*C* allele”). They also present five “*Q”* variants in almost absolute linkage disequilibrium in all human populations, occurring within a topologically associated chromatin domain (TAD): rs7095891*T, rs11003124*C, rs7084554*G, rs36014597*G, rs10556764*DelAAAGAG, and rs11003123*T. The rs7095891*T (classical “*Q”* allele) also disrupts a CpG site. Eighteen regulatory proteins bind to the DNA sequence containing this variant and the rs11003123*T allele. Among them, the forkhead box proteins FOXA1 and FOXA2 (hepatocyte nuclear factors 3 alpha and beta, respectively) and the histone acetyltransferase EP300 have the strongest affinity (ChIpSeq chromatin immunoprecipitation data from the ENCODE project, available in the UCSC Genome Browser for the HepG2 liver cell line). Interestingly, we found a trend for an association of the *LYPA* haplotype with leprosy *per se* (OR = 2.04 [95%CI = 0.98––4.28], P = 0.062), as formerly published (**Supplementary Table 2**) (25).

**Table 3:** Association of demographic and genetic variables with past/present HBV infection in leprosy patients.

Variables	Association	Univariate	Analysis		Multivariate	Analysis &		Reduced	Model		
LE HBV− *vs* HBV+	Model	OR	(95%CI)	p	OR	(95%CI)	p	OR	(95%CI)	p	P corr.
Age		1.04	(1.01−1.06)	<0.0001				1.06	(1.02−1.1)	0.002	0.008
Ethnicity		0.53	(0.26−1.1)	0.087							
MBL2*LYQC	Additive	3.49	(1.29−9.44)	0.014	**3.38**	**(1.19**−**9.57)**	**0.022**				
FCN1*1	Additive	1.68	(1.08−2.60)	0.021	**1.66**	**(1.05**−**2.63)**	**0.029**				
FCN1*3C2	Additive	0.57	(0.34−0.95)	0.03	**0.56**	**(0.33**−**0.95)**	**0.033**				
FCN2*GGGCAC	Dominant	3.53	(1.32−9.43)	0.012	**2.96**	**(1.06**−**8.25)**	**0.038**	**6.73**	**(1.66**−**27.35)**	**0.008**	**0.025**
FCN2*MAG	Dominant	0.51	(0.24−1.09)	0.084	0.62	(0.28−1.38)	0.242				
FCN2*TA	Additive	1.34	(0.89−2.02)	0.162	1.22	(0.79−1.88)	0.371				
FCN3*Del Del C	Dominant	7.74	(0.84−71.27)	0.071	**9.59**	**(1.02**−**90.34)**	**0.048**	**12.54**	**(1.16**−**135.75)**	**0.037**	**0.050**
MASP1*AC_CC	Dominant	2.88	(1.40−5.92)	0.004	**2.61**	**(1.22**−**5.58)**	**0.013**	**3.99**	**(1.31**−**12.21)**	**0.015**	**0.042**
MASP1*AC_CTG	Dominant	0.15	(0.02−1.25)	0.08	0.14	(0.02−1.20)	0.074				
MASP2*1C2-l	Dominant	3.85	(0.96−15.42)	0.057	**5.35**	**(1.22**−**23.27)**	**0.026**				
MASP2*2B1-i	Dominant	4.29	(1.29−14.24)	0.017	**4.48**	**(1.29**−**15.53)**	**0.018**	**19.25**	**(2.73**−**135.93)**	**0.003**	**0.017**
CR1*3A	Dominant	2.52	(1.24−5.14)	0.011	**2.65**	**(1.25**−**5.62)**	**0.011**				
VSIG4*TGGRCG	$	6.60	(1.36−32.05)	0.019	**10.00**	**(1.94**−**51.64)**	**0.006**	**12.55**	**(1.65**−**95.24)**	**0.014**	**0.033**
**LL HBV− *vs* HBV+**	**Model**	**OR**	**(95%CI)**	**p**	**OR**	**(95%CI)**	**p**	**OR**	**(95%CI)**	**p**	**P corr.**
Age		1.05	(1.02−1.08)	<0.0001				1.06	(1.02-1.11)	0.004	0.01
Ethnicity		0.46	(0.18−1.18)	0.107							
MBL2*HYPA	Additive	0.65	(0.36−1.16)	0.145	0.76	(0.40−1.43)	0.392				
MBL2*LYQC	Additive	3.94	(1.06−14.67)	0.041	3.47	(0.84−14.41)	0.087	**8.82**	**(1.02**−**76.59)**	**0.048**	**0.05**
FCN1*1	Additive	1.78	(1.02−3.09)	0.041	1.69	(0.93−3.07)	0.083	**2.71**	**(1.07**−**6.88)**	**0.036**	**0.03**
FCN1*3C2	Additive	0.53	(0.28−1.04)	0.064	0.61	(0.29−1.27)	0.189				
FCN2*AGAAGC	Additive	0.40	(0.18−0.89)	0.024	0.44	(0.18−1.08)	0.072				
FCN2*GGGCAC	Dominant	2.5	(0.78−8.05)	0.125	1.49	(0.42−5.31)	0.538				
FCN2*MAG	Dominant	0.23	(0.08−0.63)	0.005	**0.29**	**(0.10**−**0.86)**	**0.026**				
FCN2*TA	Additive	2.30	(1.30−4.06)	0.004	**2.15**	**(1.16**−**4.0)**	**0.015**	**2.57**	**(1.27**−**5.22)**	**0.009**	**0.02**
MASP1*AC_CC	Dominant	4.45	(1.60−12.38)	0.004	**4.99**	**(1.63**−**15.21)**	**0.005**				
MASP2*1C2-l	Dominant	6.35	(0.72−56.21)	0.096	**10.09**	**(1.01**−**100.6)**	**0.049**				
MASP2*2B1-i	Dominant	3.13	(0.58−16.82)	0.185	4.47	(0.69−29.18)	0.118	**19.71**	**(1.03**−**378.7)**	**0.048**	**0.04**
CR1*3A	Dominant	2.00	(0.82−4.88)	0.129	2.05	(0.78−5.43)	0.148				
VSIG4*TGACTA	Dominant	0.50	(0.20−1.23)	0.129	0.58	(0.21−1.65)	0.311				
**NL HBV− *vs* HBV+**	**Model**	**OR**	**(95%CI)**	**p**	**OR**	**(95%CI)**	**p**	**OR**	**(95%CI)**	**p**	**P corr.**
MBL2*HYPA	Recessive	7.09	(0.59−85.7)	0.123							
MBL2*LYQC	Dominant	3.7	(0.65−21.21)	0.142							
FCN2*GGGCAC	Dominant	6.75	(0.92−49.67)	0.061							
MASP1*AC_CC	Dominant	6.0	(1.37−26.24)	0.017				**7.15**	**(1.1**−**46.46)**	**0.039**	**0.05**
MASP2*2B1-i	Dominant	6.0	(0.87−41.44)	0.069							
CR1*3A	Additive	5.32	(1.48−19.12)	0.010				**5.06**	**(1.28**−**20.05)**	**0.021**	**0.025**

LE, Leprosy patients; LL, Lepromatous leprosy; NL, Non-lepromatous leprosy; HBV+, with past or present hepatitis B infection, as judged by positive HBsAg and/or anti-HBc and anti-HBs antigen serological results; OR, odds ratio; CI, confidence interval; p, two-tailed p value.

MBL2, mannan-binding lectin; FCN, ficolin; MASP, MBL-associated serine proteases; CR1, complement receptor; VSIG4, V-set and immunoglobulin domain containing 4.

Genetic variants to be tested in the univariate analysis were selected comparing haplotype distributions between HBV+ and HBV− individuals in any of the leprosy, lepromatous and/or non-lepromatous groups, according to a significant result of the exact Fisher test (two-tailed P value < 0.5). Only selected variables are shown.

P values equal or lower than 0.2 in the multivariate logistic regression, after correction for age and ethnicity, were used as threshold for inclusion of variables in the final model of multivariate regression.

& Corrected for age and ethnicity.

In bold: associations with genetic variants that remained significant, after correction for age and ethnicity and/or in the final reduced logistic regression model and after correction for multiple comparisons with the Benjamini−Hochberg method (P corr).

Underlined: amino acid one-letter symbols (shown in the case of missense mutations).

% considering all haplotypes associated with low MASP2 expression:

$ VSIG4 is X-linked. In this model, we considered both male and female genotypes, but did not consider as “TGGRCG” positive, female patients with heterozygous TGGRCG/TGARTA genotypes (TGARTA seems to be associated with a protective effect against leprosy per se—Stinghen et al., manuscript in preparation).

Among the seven investigated *FCN1* SNPs, all of which occur within a TAD, the combination of the most 5′ five alleles (*AAAGDelT*), present in the *3B2, *3C1, *3C2, and *3C2.3A haplotypes, occurred with higher frequency among blood donors than among HBV+ leprosy patients (OR = 0.6 [95%CI = 0.39–0.9], P = 0.014). The same protective association sustained with the addition of the next 5′ variant to the analysis (rs10117466*A), including only the summed frequencies of the *3C2 and *3C2.3A haplotypes, for the comparison between HBV− and HBV+ leprosy patients (OR = 0.58 [95%CI=0.36–0.94], P = 0.033) (**Supplementary Table 3**). Among these haplotypes, the common FCN1*3C2 haplotype presents five allelic variants associated with higher *FCN1* gene expression (rs2989727*A, rs10120023*A, rs28909976*delT, rs10117466*A and rs10858293*T) and three (rs2989727*A, rs10120023*A, rs17039495*A) that also disrupt a potentially methylated CpG site. Not surprisingly, this haplotype remained associated with resistance against HBV infection, independently of age and ethnic group distribution (OR = 0.56 [95%CI = 0.33–0.95], P = 0.033) (**Table 3**). In contrast, the frequency of the *3A.3C2.B haplotype, which presents three variants associated with lower FCN-1 expression (rs10120023*G, rs28909976*insT and rs10117466*C), was higher among HBV+ leprosy patients than among blood donors (OR = 3.64 [1.25–10.67], P = 0.023) (**Supplementary Table 3**).

There were no *FCN2* or *FCN3* haplotypes associated with HBV infection (**Supplementary Tables 4**–**6**) although we confirmed the protective association of the *FCN2* promoter-exon 1 haplotype *AGAAAC* with leprosy *per se* (OR = 0.13 [95%CI=0.02–0.99], p = 0.018) (**Supplementary Table 4**) (27).

Polymorphisms of both MBL-associated serine protease-encoding genes were associated with HBV infection. We also confirmed the recently published association with leprosy disease and the MASP1*GC_CCG haplotype, obtained with the same group of patients (OR = 1.81 [95%CI = 1.25–2.64], P = 0.002) (29). Furthermore, we identified an opposite association with the MASP1*GC_CCA haplotype, with leprosy resistance (OR = 0.50 [95%CI = 0.28–0.89], P = 0.019). The summed frequencies of MASP1*AC_CC haplotypes, diverging only at the most 3′ rs850314*G>A polymorphism, were higher among HBV+ leprosy patients compared to blood donors or HBV− leprosy patients (**Supplementary Table 6**). Susceptibility was associated with these haplotypes, independently of age and ethnicity, as well as of all other genetic associations (OR = 3.99 [95%CI = 1.31–12.21], P = 0.015) (**Table 3**).

For the *MASP2* gene, we confirmed the previously published association of the MASP2*2B1-i haplotype (regardless of the intron 4 rs2273344–intron 5 rs9430347 *AG* or *GA* combinations) with resistance against leprosy *per se* (OR = 0.29 [95%CI = 0.10–0.82], P = 0.009) (**Supplementary Table 8**) (30). In contrast, the same haplotypes were associated with susceptibility to HBV infection, independently of other genetic associations, age, and ethnic group distribution (OR = 19.25 [95%CI=2.73–135.93], P = 0.003) (**Table 3**). Furthermore, the *2B2B-l haplotype, carrying the deficiency caused by the p.Asp120Gly variant (rs72550870), was first revealed as associated with leprosy disease. This result corroborates previous observations that low MASP-2 levels may raise the risk of *M. leprae* successful infection (31) (**Supplementary Table 8**). Another MASP-2 deficiency-causing variant was also associated, but with susceptibility to HBV *per se*, independently of age and ethnicity. It substitutes arginine by histidine at the 439^th^ amino acid position within the activation site of the serine protease domain, being encoded by the *1C2-l haplotype (OR = 5.35 [95%CI = 1.22–23.27], P = 0.026) (**Table 3**).

Polymorphisms of both investigated complement receptor genes were associated with the HBV infection. Regarding *CR1*, there was a trend for a higher frequency of the *3A1 haplotype among blood donors than among HBV+ leprosy patients (OR = 2.33 [95%CI = 0.88–6.16], P = 0.087) (**Supplementary Table 9**). In fact, the summed frequencies of all *3A haplotypes were associated with increased susceptibility to HBV infection, independent of age and ethnic group distribution (OR = 2.65 (95%CI = 1.25–5.62), P = 0.011) (**Table 3**). They differ from all other *CR1* haplotypes by presenting arginine instead of histidine at the 1,208^th^ amino acid position of the CR1 protein, a missense mutation deemed as probably damaging for at least one of the *CR1* transcripts (Polyphen score of 0.98).

Although none of the X-linked *VSIG4* eQTL variants seem associated with altered gene expression/structure of *VSIG4* itself (only of neighboring genes), major haplotypes were found associated with HBV infection *per se.* The summed frequencies of two haplotypes carrying rs5964488*G (p.1208Arg), rs34581041*C, rs5964487*C, and rs9887348*G, all of which occur within TADs, were higher among HBV+ leprosy patients than among blood donors (OR = 2.11 [95%CI = 1.23–3.61], P = 0.009) and HBV− leprosy patients (OR = 1.93 [95%CI = 1.08–3.45], P = 0.034) (**Supplementary Table 10**). One of them (VSIG4*TGGRCG) remained associated with HBV infection, independent of any other associated factor (OR = 12.55 [95%CI = 1.65–95.24], P = 0.014) (**Table 3**). Conversely, frequencies of the most common *TGARTA* haplotype were lower among HBV+ leprosy patients than among blood donors (OR = 0.56 [95%CI = 0.33–0.94], P = 0.037) and HBV− leprosy patients (OR = 0.54 [95%CI = 0.30–0.91], P = 0.038) (**Supplementary Table 10**).

### Associations With Hepatitis B Virus Infection in Leprosy Patients

Three ficolin haplotypes were associated with HBV infection, restricted to the group of leprosy patients only. Although the MASP1*AC_CTG presented a trend to a leprosy-restricted HBV association as well (**Supplementary Table 7**), this was not confirmed after correction for age and ethnic group distributions (OR = 0.14 [95%CI = 0.02–1.20], P = 0.074) (**Table 3**).

Compared to FCN1*3C2 haplotypes, associated with higher *FCN1* expression and an additive effect on resistance against HBV infection *per se*, the most ancestral FCN*1 haplotype carries three major allelic variants forming CpG sites (rs2989727*G, rs10120023*G, and rs17039495*G) and at least four variants associated with lower *FCN1* gene expression (rs10120023*G, rs28909976*insT, rs10117466*C, and rs10858293*G). Not surprisingly, this haplotype was associated with higher susceptibility to leprosy/HBV infection, independently of age and ethnicity (OR = 1.66 [95%CI = 1.05–2.63], P = 0.029) (**Table 3**, **Supplementary Table 3**).

Regarding *FCN2* promoter-exon 1 haplotypes, those two sharing the first three 5′ rs3124952*G, rs3124953*G, and rs3811140*G (*GGG*) variants were also associated with increased susceptibility to leprosy/HBV infection (OR = 2.6 [95%CI = 1.13–5.96], P = 0.034) (**Supplementary Table 4**). In particular, *GGGCAC* remained associated with susceptibility to both diseases, independently of any other associated factor (OR = 6.73 [95%CI = 1.66–27.35], P = 0.008) (**Table 3**). Interestingly, the first two 5′ rs3124952*G and rs3124953*G variants of these haplotypes enhance splicing of the second exon (increasing the amount of the ENST00000350339.3 transcript, which encodes a smaller protein of 275 amino acids, presenting a shorter collagen domain). Furthermore, the last but one rs17514136*A variant within these two haplotypes is associated with lower *FCN2* expression (data from the GTEx portal). Since linkage disequilibrium is negligible between promoter-exon 1 and exon 8 *FCN2* variants, we separately evaluated the exon 8 variants encoding the fibrinogen domain. The frequencies of *TA* haplotypes (composed by rs17549193 and rs7851696 encoding the ancestral amino acids threonine at position 236 and alanine at position 258 of the ficolin-2 protein, respectively) were higher among HBV+ than among HBV− leprosy patients (OR = 2.19 [95%CI = 1.14–4.23], P = 0.022). On the other hand, *MAG* haplotypes (with methionine at position 236) were associated with protection against both diseases (OR = 0.36 [95%CI = 0.17–0.77], P = 0.011) (**Supplementary Table 5**). Although methionine and threonine seem to be equally well tolerated at amino acid position 236, the rs17549193*T variant (creating the Met codon) also disrupts a CpG site and is associated with higher *FCN2* expression. Nevertheless, exon 8 variant associations did not resist correction by age and ethnic group distribution within our setting (**Table 3**).

Lastly, the FCN3*del_del_C haplotype carrying two deletions, one in exon 5, causing a frameshift in the protein and FCN-3 deficiency (rs532781899) and another one in intron 5 (rs28362807), was associated with a dominant effect for increased susceptibility to both diseases, independent of any other associated factor (OR = 12.54 [95%CI = 1.16–135.75], P = 0.037) (**Table 3**, **Supplementary Table 6**).

### Associations With Hepatitis B Virus Infection in Lepromatous and Non-Lepromatous Patients

Despite the lack of statistical power in the two lepromatous and non-lepromatous subgroups, several of the susceptibility associations mentioned above were also found with HBV/lepromatous leprosy (MBL2*LYQC, FCN1*1, MASP1*AC_CC, MASP2*1C2-l, and MASP2*2B1-i) and with HBV/non-lepromatous leprosy (MASP1*AC_CC and CR1*3A) (**Supplementary Tables 2**, **3**, **7**–**9**, **Table 3**). The most severely affected lepromatous group also featured an opposite association with the FCN2*MAG (OR = 0.29 [95%CI = 0.10–0.86], P = 0.026) and FCN2*TA (OR = 2.57 [95%CI = 1.27–5.22], P = 0.009) haplotypes, independent of age and ethnic group distributions (**Table 3**). Nevertheless, some associations were lost after multivariate logistic correction (FCN1*3C2, FCN2*AGAAGC, and FCN2*GGGCAC with lepromatous leprosy/HBV infection and MBL2*HYPA, MBL2*LYQC, FCN2*GGGCAC with non-lepromatous leprosy/HBV infection) or due to low sample size (MASP1*AC_CTG, FCN3*2B2.2A) (**Supplementary Tables 2**–**4**, **6**, **Table 3**).

## Discussion

Evidence from growing research on HBV infection complications as liver cirrhosis, hepatocellular carcinoma, and acute-to-chronic liver failure shows a critical role for complement activation in the disease. In chronic hepatitis B patients, HBV sensitizes hepatocytes to complement-mediated killing through down-regulation of the complement-regulating membrane protein CD59, causing liver inflammation, and clearing the virus (36). Conversely, viral persistence seems associated with HBV-driven suppression of both gene expression and protein levels of complement components 2, 3, and 4 (37–40), added to lower complement activation (39). Lower gene/protein expression of these components may be understood as a simple byproduct of liver cell death and loss of healthy hepatic tissue, which is the primary source for most soluble complement proteins. Instead, we found evidence that common innate deficiency of complement proteins, manifesting either as lower serum concentrations or lower functional efficiency, predisposes to HBV infection, sometimes dependent on leprosy disease. This is the first time that the genetic susceptibility to HBV infection is investigated among leprosy patients, which are already at risk due to stigmatization and institutionalization-driven aggregation (10). From the comparison with HBV- blood donors (which unfortunately were not the same for all investigated genes, not allowing for multivariate analysis), we disentangled general associations from those particular of leprosy or even lepromatous, patients. Our approach revealed a previously unsuspected stratification within our leprosy patient group, caused by past/present HBV infection, clearly associated with institutional confinement (10). This hidden stratification may affect other patient groups as well, especially those confined to an environment that eases the spread of highly infectious diseases, as HBV.

There is a general lack of understanding regarding the role of the LP and its components and genetic variants in HBV infection and chronic disease. Most investigations refer to common *MBL2* polymorphisms, in sometimes conflicting reports starting at the late nineties (41–45). From a metanalysis including 17 studies, of which two were Brazilian (46, 47), the authors concluded that exon 1 variants leading to MBL deficiency (p.Gly54Asp, rs1800450; p.Gly57Glu, rs1800451, and p.Arg52Cys, rs5030737; known as *B, C*, and *D* variants and collectively named as *O* variants) have a dominant effect on the susceptibility to severe hepatitis B or liver cirrhosis, but not to chronic hepatitis B and hepatocellular carcinoma (48). In another Brazilian study, patients with active HBV infection and homozygous for the fully functional exon 1 MBL *A* variants presented a positive correlation between increased transaminase and HBV DNA levels and the presence of mild to moderate fibrosis (49).

In our study, we identified an association of *C*, but not of *D*, and *B-*carrying haplotypes with higher susceptibility to HBV infection. Interestingly, the *C* variant was also more frequent in non-responders to HBV vaccination in African adults (50). The reasons for an association restricted to the *LYQC* haplotypes and independent of ethnic distribution (the *C* variant is much more frequent among the African and Afro-Brazilian populations (51, 52) may be several. The deficiency associated with *O* variants is commonly taken for granted due to a profound effect on the protein’s assembly and stability, which leads to an increase of low-molecular-mass MBL that has reduced the capacity of activating complement and of ligand binding (53, 54). Nevertheless, the protein with the analogous *C* variant in rats is 10 times less efficient in activating the LP than the analogous *B* variant (55). Furthermore, the *C* variant occurs in absolute linkage disequilibrium with six so-called “*Q*” variants, which share a positive selection signature in the promoter–3′UTR region (52). Screening ENCODE ChIp-Seq data, we found 18 regulatory proteins binding to this TAD region. Those three with the highest affinity are strong hepatocyte-activating factors. This explains former findings that unequivocally associated *Q* variants with higher *MBL2* gene expression (51, 56–58). Thus, the associated *LYQC* haplotypes produce a protein with a profound defect on LP activation and probably also produce it in much higher amounts than the other *O-*harboring *LYPB* and *HYPD* (and the less common *LYPD* haplotypes) (59).

With the present study, we were also the first to demonstrate an association between *FCN1* haplotypes and HBV infection susceptibility. This gene encodes the only PRM of the LP that can be either membrane-bound or soluble, localized in gelatinase granules in the cytoplasm of neutrophils, secretory granules in monocytes, and type II alveolar epithelial cells in the lung (reviewed by 56). Haplotypes with the *AAAGDelT* combination are associated with higher *FCN1* gene expression and were associated with protection against HBV infection, particularly FCN1*3C2. On the other hand, the FCN1*3A.3C2.B haplotype and the more ancient FCN*1 haplotype were associated with lower *FCN1* gene expression and higher susceptibility to HBV and leprosy/HBV infection, respectively. This opposite association agrees with the hypothesis that the LP’s efficient activation may block HBV spread in the host.

*FCN2* polymorphisms did not emerge as associated with HBV infection itself, but only in the context of leprosy disease, indicating that this PRM probably plays a role in modulating the susceptibility to HBV within the context of the already compromised immune response of *M. leprae* infected individuals. Considering the long incubation time between *M. leprae* infection and disease manifestation decades later, this situation may be expected. In accordance, six of the same *FCN2* polymorphisms investigated in this study were also not associated with the HBV vaccinal humoral immune response (50). The susceptibility association with the promoter-exon 1 rs3124952*G, rs3124953*G, and rs3811140*G (*GGG*) combination and foremost of the *GGGCAC* haplotype is best explained by the associated differential splicing of the second exon and consequent production of a protein with a shorter collagenous domain, as well as with lower *FCN2* expression (data from the GTEx portal). Variant combinations of exon 8 (encoding the fibrinogen domain) were also associated with both diseases, but only within the lepromatous group. In this case, the ancestral amino acid sequence increased the susceptibility, whereas methionine at position 236 was associated with higher *FCN2* expression and protection. Interestingly, serum ficolin-2 concentrations were higher in chronic HBV patients than in healthy controls and HBV carriers and decreased with positive treatment response, but was lowest in individuals with hepatocellular carcinoma and cirrhosis (60).

In agreement with the previously mentioned associations with polymorphisms of PRM genes from the LP, another well-known deficiency-causing haplotype harboring the rs532781899 frameshift and causing a truncated ficolin-3 protein was associated with HBV/leprosy. This is the first time that any *FCN3* variant is associated with HBV disease. As with *FCN2* polymorphisms, the association was restricted to leprosy patients with a dominant effect, meaning that heterozygote individuals are already at higher risk. To our knowledge, the only investigation previously done revealed differential FCN-3 protein expression in HBV-related hepatocellular carcinoma (61).

Compared to the PRM-encoding genes, those encoding the LP serine proteases are highly pleiotropic, challenging the interpretation of association results. For example, *MASP1* produces at least three proteins and several predicted transcripts (Ensembl, 2020). Among the proteins, MASP-1 and MASP-3 have different catalytic domains and non-catalytic MAp44 acts as a complement regulatory protein in myocardial tissue. MASP-1 autoactivates, activates MASP-2, and cleaves C4, playing a critical role in LP activation. It is a promiscuous enzyme, also cleaving other proteins, as prothrombin in the coagulation cascade. On the other hand, MASP-3 competes with MASP-1 for the same recognition sites in the collagenous PRM stalks but cannot activate the LP. Instead, it cleaves pro-factor D from the PRM-independent alternative pathway (AP) in “resting” blood (under non-inflammatory conditions) [reviewed by (62)]. The MASP1*AC_CC haplotype associated with higher susceptibility to HBV infection presents a 3′UTR combination in exon 12 that possibly acts as a target for miRNAs, being also associated with higher MASP-3 and lower MASP-1 levels (29). Thus, the association of this *MASP1* haplotype with HBV susceptibility may be explained by blockage/hindered LP activation, as well as excessive AP activation. Interestingly, in HBV-chronically infected patients that undergo acute-on-chronic liver failure, MASP-1 production was found repressed, whereas MASP-2 was up-regulated—being both mostly produced in the liver (63).

The *MASP2* gene produces the MASP-2 serine protease and high amounts of the truncated MAp19 protein in the liver, whose function is still unclear [reviewed by (62)]. Regarding MASP-2, our results are in line with our previously published results in HIV+HBV+ individuals, whose serum levels were lower than in controls. The p.126L variant associated with MASP-2 levels <200 ng/ml increased the susceptibility to HIV+ HBV+ status (19). Interestingly, the MASP2*p.439H variant, imbedded in the *1C2-l haplotype and associated with increased susceptibility to HBV infection in this study, occurs in absolute linkage disequilibrium with p.126L. This haplotype was also formerly found associated with leprosy *per se* (30). The resulting MASP-2 protein can bind to PRMs but cannot activate. Its effect is thus much more severe than the effect of MASP2*p120G, another deficiency-causing variant that disrupts binding to PRMs since it probably reduces binding opportunities with full-working MASP-2 homodimers in heterozygotes (reviewed in 60). However, not all associations are straightforward. Some of the investigated variants’ pleiotropism, whose eQTLs mostly associate with neighboring protein-coding and lncRNA genes, cannot be overestimated. This may underly the opposite association results with MASP2*2B1-i: resistance with leprosy *per se* (as previously reported by (30) and susceptibility to HBV infection *per se*. This haplotype is related to intermediate, fully functional MASP-2 serum levels.

Both investigated complement receptor genes presented polymorphisms associated with HBV infection. Like the former genetic associations with higher susceptibility to HBV infection, probably due to genetically determined lower expression and structural protein defects, the p.1208Arg variant (rs2274567) within all CR1*3A haplotypes may alter the function of this complement receptor. However, it is still unclear if it will hinder the recognition of C3b deposited on opsonized pathogens. In agreement with our results, heterozygosity for the p.1208Arg was associated with an increased risk for HBV-related liver disease in younger men, drinkers, and non-smokers from the Guangxi Chinese population. Women with the rs3811381*G allele and heterozygous for p.1208Arg had a reduced risk of HBV-related liver cirrhosis (64).

Despite being the least studied among the genes chosen for this analysis, *VSIG4* was previously investigated for expression in HBV-infected patients. Also named as Z39Ig or CRIg, *VSIG4* encodes a macrophage complement receptor and negative regulator of T cell activation (65), whose expression is down-regulated by IFN-gamma in macrophages of patients with chronic HBV infection. Its expression is positively correlated with viral load and inversely with serum alanine aminotransaminase levels. The authors also suggested that T cells probably maintain their anti-viral function (secreting IFN-*γ*) in chronic HBV infection by down-regulating the expression of this co-inhibitory molecule (66). This gene is highly conserved, and our study is the first to investigate its polymorphisms in a candidate-gene approach. In our study, the VSIG4*TGGRCG haplotype was associated with increased susceptibility to HBV infection although none of the X-linked *VSIG4* eQTL variants within this haplotype seems associated with altered *VSIG4* gene expression/structure (GTEx Portal). The opposite association between two relatively common *VSIG4* haplotypes that differ in three polymorphic positions—*TGARTA* (protective) and *TGGRCG* (susceptibility) seem to rely chiefly on the two last polymorphisms, which occur within an enhancer region (data from the ENCODE project, UCSC Genome Browser).

This is the most thorough and complete investigation done with genetic polymorphisms of complement components and HBV infection. However, our work does not compare with others who evaluated the role of some of these components on HBV chronification and severe disease since most of the leprosy patients already resolved viral infection at the time of sampling. Furthermore, patient samples were collected in 2002. During that year, HBV detection by real-time quantitative PCR started to get recommended as a diagnostic and prognostic tool in the literature [reviewed by (67)] but was still not established for clinical practice. In our setting, HBV quantification would have been of value for five individuals with active HBV infection, as well as for the detection of a possible occult HBV infection (68). In practice, this would nevertheless leave our results unchanged since we checked for associations with HBV infection *per se*, not active/past acute/chronic infection or its complications.

Moreover, the almost complete absence of current HBV infection (determined by positive HBsAg) among this study’s participants is also the most plausible reason for the lack of association with serum levels of the investigated soluble proteins. Thus, the presence of HBV positive serum markers was not accompanied by an inflammatory response at sampling time. Furthermore, the fact that the evaluated *C3* polymorphisms were not associated in this setting does not exclude its importance in the anti-viral immunological response, as mentioned before. Sample size, especially in the lepromatous and non-lepromatous groups, limited the statistical power of this study. Thus, exclusive results from these groups should be cautiously evaluated. Finally, although we found substantial support for functional causality of the associated polymorphisms and gene expression/structure, studies confirming their role in the modulation of HBV infection and HBV/leprosy coinfection are still missing, and the reported associations must be replicated in other settings, to be confirmed.

**Figure 1:**
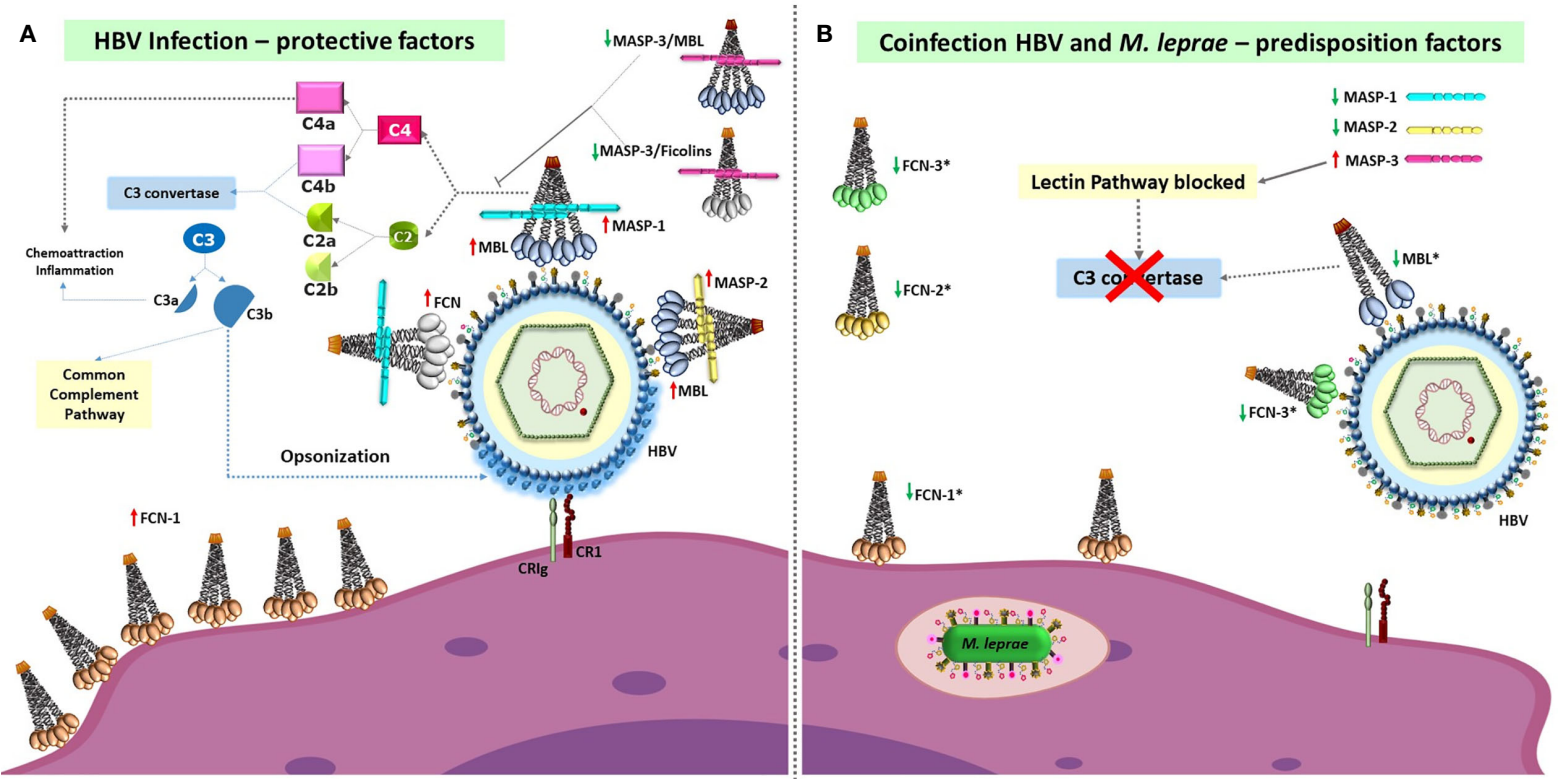
Susceptibility and protective factors of the lectin pathway of complement and complement receptors, for HBV infection *per se* and HBV infection/leprosy. **(A)** High levels of fully functional proteins from the lectin pathway (with the possible exception of MASP-3), and probably also of complement receptors (CR1 and CRIg, officially VSIG4), are necessary to defeat HBV infection through complement activation and opsonophagocytosis. Recognition of HBV-associated molecular patterns by MBL and FCNs associated with MASPs causes MASP-1 autoactivation, MASP-1-driven transactivation of MASP-2 (complexed to another MBL or FCN oligomer), cleavage of C4 and C2, formation of the C3 convertase and cleavage of C3, covering the virus with C3b opsonins. These are recognized by CR1 and CRIg complement receptors, leading to phagocytosis and viral destruction. FCN-1 may also be presented on the phagocyte cell surface through recognition of self-sialic acid residues, but its function as a membrane receptor remains elusive. **(B)** In contrast, low levels of pattern-recognition receptors of the lectin pathway (marked with an asterisk), as well as of other complement proteins, are associated with higher susceptibility to HBV infection and leprosy/HBV infection (represented by *Mycobacterium leprae* within the phagolysosome). The associated polymorphisms cause: dysfunctional MBL, FCN-3 and MASP-2 molecules (given by the p.57E “*C*” variant in MBL, the frameshift caused by the *+1637* deletion in FCN-3, and p.439H variant in MASP-2), lower MASP-1 levels and higher MASP-3 levels (associated with the MASP1*AC_CC haplotype), lower FCN-2 serum levels due to preferential alternative splicing of exon 2 and production of higher amounts of FCN-2 proteins with a shorter collagenous tail (associated with the FCN2*GGGCAC haplotype), and lower levels of functional FCN-1 levels (associated with eQTLs of the FCN1*1 haplotype). These alterations are expected to greatly reduce activation of the lectin complement pathway or to inhibit it (*e.g.*, due to higher MASP-3 levels, as well as dysfunctional MASP-2 molecules). Furthermore, the p.1208Arg encoded by CR1*3A haplotypes and CRIg molecules encoded by VSIG4*TGGRCG increase susceptibility to the disease, probably by affecting internalization of the virus. MBL, Mannan-binding lectin; FCN, ficolin; MASP, MBL-associated serine protease; CR1, complement receptor 1; CRIg, complement receptor immunoglobulin, encoded by VSIG4 (V-set and immunoglobulin domain containing 4). Red upward-pointing arrows: high/normal expression of functional MASP homodimers/MBL/FCN oligomers. Green downward-pointing arrows: low expression of functional MASP homodimers/MBL/FCN oligomers (sometimes accompanied by high levels of dysfunctional molecules, forming dimers/trimers that do not complex with MASP molecules).

This work is also unique in the retrospective approach of comparing leprosy patients who have had/had not HBV infection and identifying possible genetic variants whose effects manifest particularly within the leprosy-affected group by comparing the results with those obtained with HBV positive patients and blood donors. Furthermore, the associations found for HBV infection or leprosy/HBV infection were, with few exceptions, not the same as formerly found for leprosy *per se*, agreeing with different roles probably played by complement on the routes used by HBV and *M. leprae* for successful infection and to induce disease (25–31). Finally, our results lead us to suggest that HBV infection occurs more often in individuals carrying common variants that affect protein structure or induce low levels of LP components, decreasing the activity of the proteolytic cascade and possibly phagocytic activity (**Figure 1**). Furthermore, ficolin gene polymorphisms represent another layer of genetic predisposition, modulating the susceptibility to HBV infection in leprosy patients. The associations revealed in this setting lead us to suggest a critical role of the LP and complement receptors in controlling susceptibility to HBV infection, an association maintained and even reinforced within the context of leprosy disease.

## Data Availability Statement

The datasets presented in this study can be found in online repositories. The names of the repository/repositories and accession number(s) can be found in the article/**Supplementary Material**.

## Ethics Statement

The studies involving human participants were reviewed and approved by the Medical ethics committee of the HC-UFPR. The patients/participants provided their written informed consent to participate in this study.

## Author Contributions

IM administered the project and supervised this work. AB contributed to the conception of the work, and curated and analyzed the data. AM obtained and prepared the samples. IM, AB, HM, GK, STS, FA, VB, and LG performed the investigation. AB, HM, SS, FA, LG, and GK further provided methodological input by developing the multiplex PCR-SSP methods for genotyping. ES provided the samples, and IM, TV, and ST provided resources for the analysis. IM, ST, and TV acquired the funding. AB and CO drafted and edited the manuscript, after critical review for intellectual content, by all coauthors. All authors contributed to the article and approved the submitted version.

## Funding

This work was supported by the 01/2007 and 518/2010 PRODOC grants of CAPES (Coordenação de Aperfeiçoamento de Pessoal Superior, http://www.capes.gov.br/bolsas/bolsasno-pais/prodoc) for AB and by the 034/2008 CNPq (Conselho Nacional de Desenvolvimento Científico e Tecnológico, http://www.cnpq.br/web/guest/bolsas-e-auxilios) and Fundação Araucária (http://www.fappr.pr.gov.br/) grants for IM. We thank the Coordenação de Aperfeiçoamento de Pessoal de Nível Superior (CAPES/PROAP–Finance Code 001) for financial support and for the scholarships provided to GK (CAPES-40001016006P1), VB (CAPES-40001016006P1), CT (CAPES-40001016012P1), and HM (CAPES-40001016012P1). LG receives a CNPq scholarship (141955/2020-1). AB currently receives a research productivity scholarship from CNPq (314288/2018-0). She also received a CAPES-PRINT scholarship (41/2017) to enable the collaboration with TV from the Tübingen University, also fostering the collaboration with ST from the Aarhus University. FA was a PNPD (Programa Nacional de Pós Doutorado)/CAPES post-doc fellow. ST was funded by the Danish Research Council. The funders had no role in the study design, data collection and analysis, decision to publish, or preparation of the manuscript.

## Conflict of Interest

The authors declare that the research was conducted in the absence of any commercial or financial relationships that could be construed as a potential conflict of interest.

## Supplementary Material

Supplementary Figure 1 Distribution of serum levels of complement components in HBV+ (white) and HBV− (gray) leprosy patients. Sample sizes for each measured component are listed in **Table 2**. Box-whisker plots depict 10th, 25th, 50th, 75th and 90th percentiles.
