# Supplementary figures and images for: Hepatitis B Virus Infection Among Leprosy Patients: A Case for Polymorphisms Compromising Activation of the Lectin Pathway and Complement Receptors
Source: Front Immunol. 2021 Feb 11;11:574457. doi: 10.3389/fimmu.2020.574457 (PMC7904891; doi:10.3389/fimmu.2020.574457)

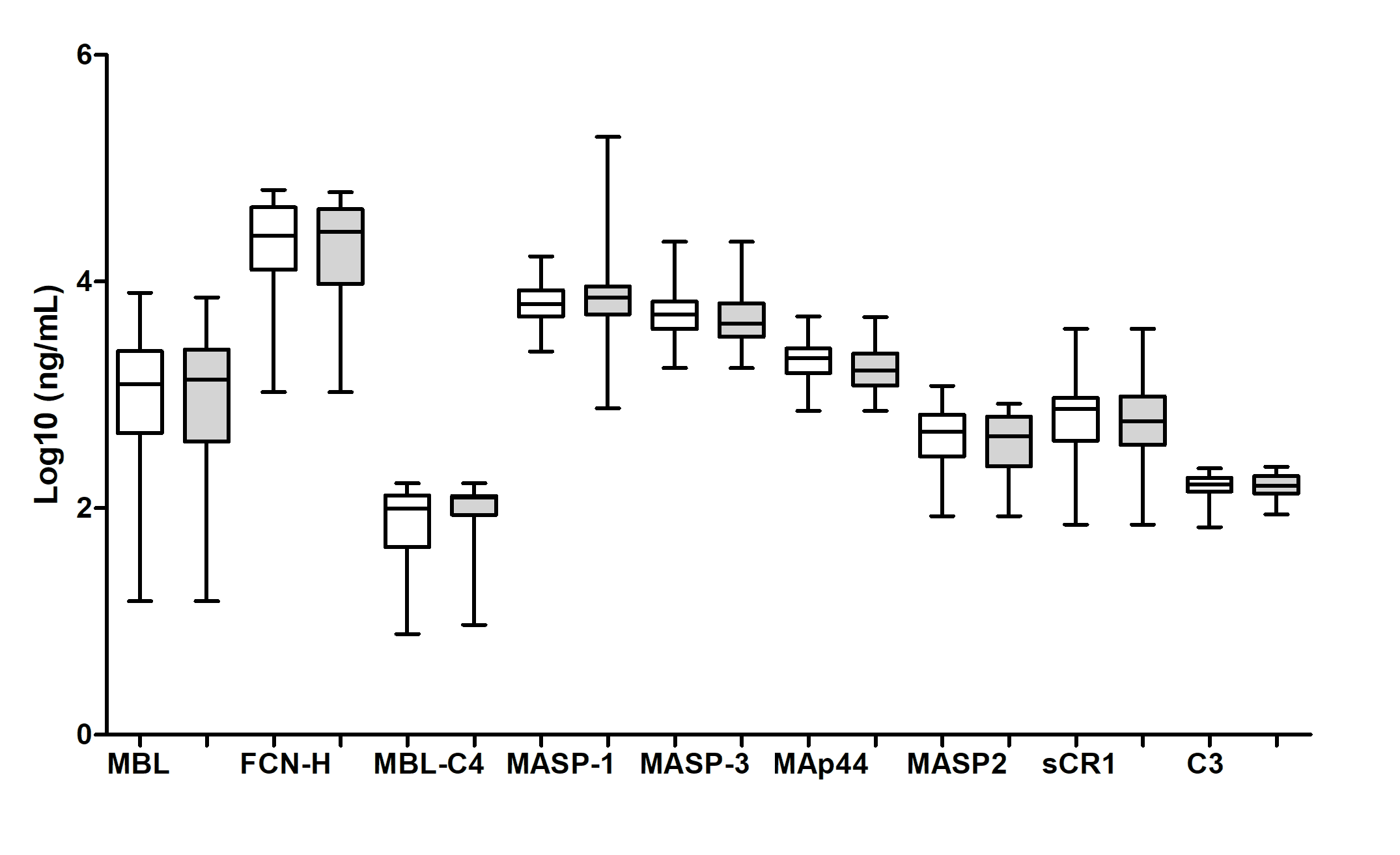

Supplement: Supplementary Figure 1 — Distribution of serum levels of complement components in HBV+ (white) and HBV− (gray) leprosy patients. Sample sizes for each measured component are listed in Table 2. Box-whisker plots depict 10th, 25th, 50th, 75th and 90th percentiles. [file Image_1.tif]
