# Supplementary material for: Hepatitis B Virus Infection Among Leprosy Patients: A Case for Polymorphisms Compromising Activation of the Lectin Pathway and Complement Receptors
Source: Front Immunol. 2021 Feb 11;11:574457. doi: 10.3389/fimmu.2020.574457 (PMC7904891; doi:10.3389/fimmu.2020.574457)
Supplement: Supplementary file 2 [file Table_1.docx]

Supplementary Material

# Supplementary Table 1. *VSIG4* and *C3* sequence-specific primer sequences.

| **Gene region** | **Position** | **SNP** | **Direction** | **5’-3’ Sequence** | **Fragment (bp)** |
| --- | --- | --- | --- | --- | --- |
| *VSIG4* promoter | -1431 | rs2284705 | Fwd | TGG TGT AGT GGT GAT AGG GTG **T** | 792 |
|  |  |  |  | TGG TGT AGT GGT GAT AGG GTG **A** |  |
|  | -685 | rs5964489 | Rev | TCA CTT TCT ACA ATG GCT CCA CCT **C** |  |
|  |  |  |  | TCA CTT TCT ACA ATG GCT CCA CCT **G** |  |
| *VSIG4 i*ntron 1 | +6069 | rs5964488 | Fwd | GTG ATA GAA AGA AAG TTG AGT GGC **A** | 412 |
|  |  |  |  | GTG ATA GAA AGA AAG TTG AGT GGC **G** |  |
| *VSIG4* exon 2 | +6432 | rs34581041 | Rev | TGA CTT CAC ACG TGT AGT GGC TCC **G** |  |
|  |  |  |  | TGA CTT CAC ACG TGT AGT GGC TCC **A** |  |
| *VSIG4* intron 3 | +10234 | rs5964487 | Fwd | CCT CAA ACC TGT TAG AAA ACA **T** | 768 |
|  |  |  |  | CCT CAA ACC TGT TAG AAA ACA **C** |  |
|  | +10899 | rs9887348 | Rev | GAC ATG AGA AAA CCA TAA GCT **C** |  |
|  |  |  |  | GAC ATG AGA AAA CCA TAA GCT **T** |  |
| *C3* intron 2 | -111 | rs2250656 | Fwd | AAA ACG GCC ACC TCG G**A** | 182 |
|  |  |  |  | AAA ACG GCC ACC TCG G**G** |  |
| *C3* exon 3 | 37 | rs2230199 | Rev | CGG TCA CGA ACT TGT TGC **G** |  |
|  |  |  |  | CGG TCA CGA ACT TGT TGC **C** |  |
| *C3* exon 9 | 65 | rs1047286 | Fwd | GAC GGG GTG CAG AAC C**C** | 752 |
|  |  |  |  | GAC GGG GTG CAG AAC C**T** |  |
| *C3* exon 10 |  |  | Rev | CAA AGG GCA TTC CTG GTT TGA |  |

*VSIG4* - V-set and immunoglobulin domain containing 4, *C3* – complement component 3, SNP – single nucleotide polymorphism.

Fwd – forward, Rev – reverse, SNP – single nucleotide polymorphism, bp - base pairs.

In bold: variant nucleotide

For each sample, four PCR-SSP reactions were necessary to haplotype two SNPs (excepting *C3* exon 9 – exon 10). For each PCR reaction, 50 to 100 ng of genomic DNA were used. The reaction solution was prepared with 1x Coral buffer (Invitrogen Life Technologies, Carlsbad, CA, United States), 1.5% glycerol, 0.5 mM MgCl2, 0.2 mM triphosphate deoxynucleotide (dNTP), 0.2-0.3 uM primers (Eurofins MWG Operon, CA, United States), 0.03 U/uL Taq DNA polymerase (Invitrogen Life Technologies, Carlsbad, CA, USA). To amplify the *G* allele of *VSIG4* intron 1_ + 6069, +6432, the Q solution (Invitrogen Life Technologies, Carlsbad, CA, USA) was used to increase the specificity of the reaction. PCR-SSP was performed in a T100 ™ Thermal Cycler thermocycler (Bio-Rad, CA, United States) in 30-35 cycles, denaturation temperature of 94ºC for 20 seconds, and extension temperature of 72ºC for 30 seconds. In all PCR reactions, endogenous controls, positive controls and negative controls were included, which are complete reaction solutions without the DNA. To control the PCR reaction, a specific endogenous control was used, which consists of a pair of generic primers, compatible with the PCR-SSP primers, that amplify a specific non-variable sequence of a gene. The endogenous control must amplify in all samples tested and in the positive controls to validate a PCR reaction. The positive control consists of a sample with a known genotype, being used to control for efficient genotyping. The annealing temperatures were different for each reaction, with a decrease in temperature every 10-15 cycles (PCR touchdown) to increase specificity and sensitivity, as described below for *VSIG4*: Promotor_-1431, -685: 10 cycles at 66ºC, 10 cycles at 64ºC, 10 cycles at 62ºC; Intron1 / Exon2_ + 6069, +6432: 10 cycles at 70ºC, 15 cycles at 68ºC, 10 cycles 66ºC; Intron3_ + 10234, + 10899: 10 cycles at 65ºC, 10 cycles at 63ºC, 15 cycles at 61ºC. The C3-rs2250656A_For or C3-rs2250656G_For primers were conjugated to C3-rs2230199C_Rev or rs2230199G_Rev primers to generate a 182bp C3 fragment, while the C3-rs1047286C_For or C3-rs1047286T_For were conjugated to C3-exon10_Rev primer to generate a 752 bp fragment. Simultaneously a 1059 bp control fragment of the *MBL2* gene was amplified (MBL_For: ATGGGGCTAGGCTGCTGAG; MBL_Rev CCAACACGTACCTGGTTCCC). The amplification protocol started with a 5 minute denaturation step at 94 C, followed by 10 cycles of 20s at 94 C, 30s at 64 C and 30s at 72 C; 10 cycles of 20s at 94 C, 30s at 62 C and 30s at 72C; 10 cycles of 20s at 94 C, 30s at 60 C and 30s at 72 C, concluding with 5 min at 72 C in the final DNA extension step. The result was observed after electrophoretic run on SYBR® Safe stained 1.5% agarose gel (Invitrogen).
