# Supplementary material for: Hepatitis B Virus Infection Among Leprosy Patients: A Case for Polymorphisms Compromising Activation of the Lectin Pathway and Complement Receptors
Source: Front Immunol. 2021 Feb 11;11:574457. doi: 10.3389/fimmu.2020.574457 (PMC7904891; doi:10.3389/fimmu.2020.574457)
Supplement: Supplementary file 3 [file Table_2.docx]

Supplementary Material

# Supplementary Table 2. Distribution of MBL2 haplotypes in leprosy patients, according to HBV infection and severity of leprosy disease (lepromatous or not).

| *MBL2* |  | Promoter –  exon 1 | Co |  | OR | p | LE |  | LE |  | OR | p | LL |  | LL |  | OR | p | NL |  | NL |  | OR | p |
| --- | --- | --- | --- | --- | --- | --- | --- | --- | --- | --- | --- | --- | --- | --- | --- | --- | --- | --- | --- | --- | --- | --- | --- | --- |
| Haplotype | # | Sequence | HBV- |  | (95%CI) |  | HBV- |  | HBV+ |  | (95%CI) |  | HBV- |  | HBV+ |  | (95%CI) |  | HBV- |  | HBV+ |  | (95%CI) |  |
|  | N |  | 400 | % |  |  | 232 | % | 148 | % |  |  | 126 | % | 106 | % |  |  | 106 | % | 42 | % |  |  |
| **1A1-h* | *LYPA* | *CGCCRGG* | 18 | 4.5 | 2.04 | 0.062 | 18 | 7.76 | 13 | 8.78 |  |  | 7 | 5.56 | 12 | 11.32 |  |  | 11 | 10.38 | 2 | 4.76 |  |  |
|  |  |  |  |  | (0.98-4.28) |  |  |  |  |  |  |  |  |  |  |  |  |  |  |  |  |  |  |  |
| ****1B1-h*** | ***HYPA*** | ***GGCCRGG*** | 122 | 30.5 |  |  | 67 | 28.88 | 44 | 29.73 |  |  | 42 | 33.33 | 26 | 24.53 |  |  | 25 | 23.58 | 18 | 42.86 | **2.43** | **0.027** |
|  |  |  |  |  |  |  |  |  |  |  |  |  |  |  |  |  |  |  |  |  |  |  | **(1.14-5.19)** |  |
| **1B2-l* | *HYPD* | *GGCCCGG* | 26 | 6.5 | 0.30 | 0.05 | 10 | 4.31 | 3 | 2.03 |  |  | 6 | 4.76 | 3 | 2.83 |  |  | 4 | 3.77 | 0 | 0 |  |  |
|  |  |  |  |  | (0.09-1.0) |  |  |  |  |  |  |  |  |  |  |  |  |  |  |  |  |  |  |  |
| **1B3* | *HYPG* | *GGCTRGG* | 0 | 0 |  |  | 1 | 0.43 | 0 | 0 |  |  | 0 | 0 | 0 | 0 |  |  | 1 | 0.94 | 0 | 0 |  |  |
| **1C1-l* | *LXPA* | *CCCCRGG* | 71 | 17.75 |  |  | 35 | 15.09 | 20 | 13.51 |  |  | 15 | 11.90 | 16 | 15.09 |  |  | 20 | 18.87 | 4 | 9.52 |  |  |
| **1F1-l* | *LYPB* | *CGCCRDG* | 64 | 16 |  |  | 43 | 18.53 | 21 | 14.19 |  |  | 23 | 18.25 | 15 | 14.15 |  |  | 20 | 18.87 | 6 | 14.29 |  |  |
| **1F2-l &* | *LYPB* | *CGCCRDG* | 0 | 0 |  |  | 0 | 0 | 1 | 0.68 |  |  | 0 | 0 | 0 | 0 |  |  | 0 | 0 | 1 | 2.38 |  |  |
| **1H1-h &* | *LYPA* | *CGCCRGG* | 7 | 1.75 |  |  | 5 | 2.16 | 2 | 1.35 |  |  | 5 | 3.97 | 1 | 0.94 |  |  | 0 | 0 | 1 | 2.38 |  |  |
| **1J1-h &* | *LYPA* | *CGCCRGG* | 0 | 0 |  |  | 2 | 0.86 | 1 | 0.68 |  |  | 1 | 0.79 | 0 | 0 |  |  | 1 | 0.94 | 1 | 2.38 |  |  |
| **4A1-h* | *LYQA* | *CGTCRGG* | 78 | 19.5 |  |  | 42 | 18.10 | 30 | 20.27 |  |  | 24 | 19.05 | 23 | 21.70 |  |  | 19 | 17.92 | 7 | 16.67 |  |  |
| **4C1-h &* | *LYQA* | *CGTCRGG* | 2 | 0.5 |  |  | 0 | 0 | 0 | 0 |  |  | 0 | 0 | 0 | 0 |  |  | 0 | 0 | 0 | 0 |  |  |
| ****4F1-l*** | ***LYQC*** | ***CGTCRGE*** | 8 | 2 | **3.76 $** | **0.003** | 4 | 1.72 | 8 | 5.41 | **3.63** | **0.014** | 2 | 1.59 | 7 | 6.6 | **4.27** | **0.023** | 2 | 1.89 | 0 | 0 |  |  |
|  |  |  |  |  | **(1.61-8.76)** |  |  |  |  |  | **(1.35-9.77)** |  |  |  |  |  | **(1.14-15.95)** |  |  |  |  |  |  |  |
| ****4F2A-l &*** | ***LYQC*** | ***CGTCRGE*** | 2 | 0.5 |  |  | 2 | 0.86 | 3 | 2.03 |  |  | 1 | 0.79 | 2 | 1.89 |  |  | 1 | 0.94 | 1 | 2.38 |  |  |
| ****4F3-l &*** | ***LYQC*** | ***CGTCRGE*** | 0 | 0 |  |  | 0 | 0 | 2 | 1.35 |  |  | 0 | 0 | 1 | 0.94 |  |  | 0 | 0 | 1 | 2.38 |  |  |
| **4E1-h &* | *LYQA* | *CGTCRGG* | 1 | 0.25 |  |  | 3 | 1.29 | 0 | 0 |  |  | 0 | 0 | 0 | 0 |  |  | 2 | 1.89 | 0 | 0 |  |  |
| **1A1.1B2-l* | *LYPD* | *CGCCRDG* | 1 | 0.25 |  |  | 0 | 0 | 0 | 0 |  |  | 0 | 0 | 0 | 0 |  |  | 0 | 0 | 0 | 0 |  |  |

*MBL2* – Mannan-binding lectin. N = number of chromosomes

LE – Leprosy patients, LL – Lepromatous leprosy, NL – Non-lepromatous leprosy.

HBV+ - with past or present hepatitis B infection, as judged by positive anti-HBc or HBsAg sorological results, respectively.

OR – odds ratio, CI – confidence interval, p – two-tailed p value. In bold: significant difference for haplotype frequencies, obtained with the exact Fisher’s test (only results with p values < 0.1 are given). Underlined: aminoacid one-letter symbols (shown in the haplotype sequence, in the case of missee mutations)

$ Association with *LYQC* haplotypes (**4F1-l, *4F2A-l* and **4F3-l*)

The following polymorphisms compose common *MBL2* haplotypes (in order of appearance in the [NC_000010](https://www.ensembl.org/Homo_sapiens/Location/View?contigviewbottom=variation_feature_variation%3Dnormal;db=core;source=dbSNP;v=rs11003125;vdb=variation;vf=26951267).11 reference sequence, preceded by their common name and with the corresponding nucleotides, within parentheses): *H/L* variant: *g.52772254G>C,* rs11003125 (*H=G/L=C*);  *X/Y* variant: *g.52771925G>C*, rs7096206 (*X=C/Y=G*); *P/Q* variant: *g.52771701 G>A,* rs7095891 (*P=C/Q=T*)*; A/G* variant: *g.52771504G>A,* p.Asn44=, rs34120190 (*A*=*C/G=T*); *A/D* variant: *g.52771482G>A,* p.Arg52Cys, rs5030737 (*A=C/D=T*); *A/B* variant: *g.52771475C>T,* p.Gly54Asp, rs1800450 (*A=G/B=A*); *A/C* variant: *g.52771466C>T,* p.Gly57Glu, rs1800451 (*A*=*G/C=A*). The *P/Q* variant occurs in strong linkage disequilibrium with rs11003124 (*A/C*)*,* rs7084554 *(A/G*)*,* rs36014597 *(A/G*)*,* rs10556764 *(indelAAAGAG*) and rs11003123 (*C/T*).

& Other variants included in “non-classical” *MBL2* haplotypes:

**1F2-l* : *g.52771739A>G*, rs72661131 (*T/C*, disrupts a TATA box)

**1H1-h*: *g.52772139C>T*, rs7100749 (*G/A*)

**1J1-h*: *g.52772049C>T*, rs72661122 (*G/A*)

**4C1-h* : *g.52772071C>A*, rs35615810 (*G/T*)

**4F2A-l*: *g.52771730G>T*, rs45602536 (*C/A*)

**4F3-l*: *g.52771815T>A,* rs67990116 (*A/T*)

**4E1-h: g.52771949C>T*, rs35236971 (*G/A*)

# Haplotype nomenclature widely used since proposed in the nineties, updated with a phylogenetic nomenclature (in the first column) by (45).
