# Supplementary material for: Hepatitis B Virus Infection Among Leprosy Patients: A Case for Polymorphisms Compromising Activation of the Lectin Pathway and Complement Receptors
Source: Front Immunol. 2021 Feb 11;11:574457. doi: 10.3389/fimmu.2020.574457 (PMC7904891; doi:10.3389/fimmu.2020.574457)
Supplement: Supplementary file 4 [file Table_3.docx]

Supplementary Material

# Supplementary Table 3. Distribution of *FCN1* haplotypes in leprosy patients, according to HBV infection and severity of leprosy disease (lepromatous or not).

| *FCN1* | Promoter | Co |  | OR | p | LE |  | LE |  | OR | p | LL |  | LL |  | OR | p | NL |  | NL |  |
| --- | --- | --- | --- | --- | --- | --- | --- | --- | --- | --- | --- | --- | --- | --- | --- | --- | --- | --- | --- | --- | --- |
| Haplotype # | Sequence | HBV- |  | (95%CI) |  | HBV- |  | HBV+ |  | (95%CI) |  | HBV- |  | HBV+ |  | (95%CI) |  | HBV- |  | HBV+ |  |
| N |  | 698 | % |  |  | 224 | % | 148 | % |  |  | 124 | % | 106 | % |  |  | 100 | % | 42 | % |
| ****1*** | ***GAGGInsTCG*** | 256 | 36.68 |  |  | 73 | 32.6 | 66 | 44.6 | **1.66** | **0.021** | 42 | 33.9 | 50 | 47.2 | **1.74** | **0.044** | 31 | 31 | 16 | 38.1 |
|  |  |  |  |  |  |  |  |  |  | **(1.09-2.55)** |  |  |  |  |  | **(1.02-2.97)** |  |  |  |  |  |
| **2* | *GGGGInsTCG* | 29 | 4.15 |  |  | 8 | 3.57 | 6 | 4.05 |  |  | 5 | 4.03 | 4 | 3.77 |  |  | 3 | 3 | 2 | 4.76 |
| **3A* | *AAGGInsTCG* | 18 | 2.58 |  |  | 8 | 3.57 | 2 | 1.35 |  |  | 3 | 2.42 | 1 | 0.94 |  |  | 5 | 5 | 1 | 2.38 |
| **3B1* | *AAGGDelTCG* | 160 | 22.92 |  |  | 57 | 25.4 | 35 | 23.6 |  |  | 32 | 25.8 | 26 | 24.5 |  |  | 25 | 25 | 9 | 21.4 |
| ****3B2*** | ***AAAGDelTCG*** | 2 | 0.29 | **0.60 $** | **0.014** | 0 | 0 | 0 | 0 |  |  | 0 | 0 | 0 | 0 |  |  | 0 | 0 | 0 | 0 |
|  |  |  |  | **(0.39-0.90)** |  |  |  |  |  |  |  |  |  |  |  |  |  |  |  |  |  |
| ****3C1*** | ***AAAGDelTCT*** | 20 | 2.87 |  |  | 2 | 0.89 | 1 | 0.68 |  |  | 1 | 0.81 | 1 | 0.94 |  |  | 1 | 1 | 0 | 0 |
| ****3C2*** | ***AAAGDelTAT*** | 201 | 28.80 | 0.68 | 0.085 | 71 | 31.7 | 32 | 21.6 | **0.59** | **0.034** | 36 | 29 | 20 | 18.9 | 0.57 | 0.09 | 35 | 35 | 12 | 28.6 |
|  |  |  |  | (0.45-1.04) |  |  |  |  |  | **(0.37-0.96)** |  |  |  |  |  | (0.31-1.06) |  |  |  |  |  |
| ****3A.3C2.B*** | ***AAGAInsTCT*** | 8 | 1.15 | **3.64** | **0.023** | 3 | 1.34 | 6 | 4.05 |  |  | 3 | 2.42 | 4 | 3.77 |  |  | 0 | 0 | 2 | 4.76 |
|  |  |  |  | **(1.25-10.67)** |  |  |  |  |  |  |  |  |  |  |  |  |  |  |  |  |  |
| **3A.3C2.A* | *AAGGInsTCT* | 0 | 0 |  |  | 1 | 0.45 | 0 | 0 |  |  | 1 | 0.81 | 0 | 0 |  |  | 0 | 0 | 0 | 0 |
| ****3C2.3A*** | ***AAAGDelTAG*** | 4 | 0.57 | 0.66 & | 0.069 | 1 | 0.45 | 0 | 0 | **0.58 &** | **0.033** | 1 | 0.81 | 0 | 0 |  |  | 0 | 0 | 0 | 0 |
|  |  |  |  | (0.43-1.01) |  |  |  |  |  | **(0.36-0.94)** |  |  |  |  |  |  |  |  |  |  |  |

*FCN1 -* ficolin 1. N = number of chromosomes

LE – Leprosy patients, LL – Lepromatous leprosy, NL – Non-lepromatous leprosy.

HBV+ - with past or present hepatitis B infection, as judged by positive anti-HBc or HBsAg sorological results, respectively.

OR – odds ratio, CI – confidence interval, p – two-tailed p value. In bold: significant difference for haplotype frequencies, obtained with the exact Fisher’s test (only results with p values < 0.1 are given). # Phylogenetic nomenclature published by (19).

$ Association with *AAGDelT...* haplotypes (**3B2, *3C1, *3C2, *3C2.3A*)

& Association with **3C2* haplotypes containing the -*144*A* variant (**3C2* and **3C2.3A*).

The following polymorphisms compose *FCN1* haplotypes (in order of appearance in the [NC_000009](https://www.ensembl.org/Homo_sapiens/Location/View?contigviewbottom=variation_feature_variation%3Dnormal;db=core;source=dbSNP;v=rs2989727;vdb=variation;vf=686347875).12 reference sequence, preceded by their common name and with the corresponding nucleotides, within parentheses): *-1981* variant: *g.134919852C>T,* rs2989727 (*A/G*); *-791* variant: *g.134918662T>C*, rs28909068 (*A/G*); *-541* variant: *g.134918413C>T,* rs10120023 (*G/A*); *-399* variant: *g.134918270C>T,* rs17039495 (*G/A*); *-271* variant: *g.134918143del,* rs28909976 (*indelT*); *-144* variant: *g.134918015G>T,* rs10117466 *(C/A*); *+33* variant: *g.134917839C>A,* rs10858293 (*G/T*)*.*
