# Supplementary material for: Hepatitis B Virus Infection Among Leprosy Patients: A Case for Polymorphisms Compromising Activation of the Lectin Pathway and Complement Receptors
Source: Front Immunol. 2021 Feb 11;11:574457. doi: 10.3389/fimmu.2020.574457 (PMC7904891; doi:10.3389/fimmu.2020.574457)
Supplement: Supplementary file 5 [file Table_4.docx]

Supplementary Material

# Supplementary Table 4. Distribution of *FCN2* promoter – exon 1 haplotypes in leprosy patients, according to HBV infection and severity of leprosy disease (lepromatous or not).

| *FCN2* | Promoter - exon 1 | Co |  | OR | p | LE |  | LE |  | OR | p | LL |  | LL |  | OR | p | NL |  | NL |  |
| --- | --- | --- | --- | --- | --- | --- | --- | --- | --- | --- | --- | --- | --- | --- | --- | --- | --- | --- | --- | --- | --- |
| Haplotype # | Sequence | HBV- |  | (95%CI) |  | HBV- |  | HBV+ |  | (95%CI) |  | HBV- |  | HBV+ |  | (95%CI) |  | HBV- |  | HBV+ |  |
| N |  | 262 | % |  |  | 166 | % | 112 | % |  |  | 90 | % | 82 | % |  |  | 76 | % | 30 | % |
| h1 | *AAAAAC* | 48 | 18.32 |  |  | 32 | 19.3 | 20 | 17.9 |  |  | 18 | 20 | 16 | 19.5 |  |  | 14 | 18.4 | 4 | 13.3 |
| h2 | ***AGAAAC* $** | 17 | 6.49 | **0.13** | **0.018** | 2 | 1.2 | 1 | 0.89 |  |  | 2 | 2.22 | 1 | 1.22 |  |  | 0 | 0 | 0 | 0 |
|  |  |  |  | **0.02-0.99** |  |  |  |  |  |  |  |  |  |  |  |  |  |  |  |  |  |
| h3 | ***AGAAGC*** | 63 | 24.05 |  |  | 45 | 27.1 | 25 | 22.3 |  |  | 28 | 31.1 | 14 | 17.1 | **0.46** | **0.035** | 17 | 22.4 | 11 | 3.67 |
|  |  |  |  |  |  |  |  |  |  |  |  |  |  |  |  | **(0.22-0.94)** |  |  |  |  |  |
| h4 | *GGAAAC* | 113 | 43.13 |  |  | 73 | 44 | 46 | 41.1 |  |  | 35 | 38.9 | 36 | 43.9 |  |  | 38 | 50 | 10 | 33.3 |
| h5 | *GGAAAT* | 4 | 1.53 |  |  | 3 | 1.81 | 4 | 3.57 |  |  | 1 | 1.11 | 4 | 4.88 |  |  | 2 | 2.6 | 0 | 0 |
| h6 | ***GGGCAC*** | 15 | 5.73 |  |  | 7 | 4.22 | 15 | 13.4 | **3.51** | **0.007** | 5 | 5.56 | 11 | 13.4 |  |  | 2 | 2.6 | 4 | 13.3 |
|  |  |  |  |  |  |  |  |  |  | **(1.38-8.91)** |  |  |  |  |  |  |  |  |  |  |  |
| h7 | ***GGGAAC*** | 2 | 0.76 |  |  | 3 | 1.81 | 1 | 0.89 | **2.6 &** | **0.034** | 1 | 1.11 | 0 | 0 |  |  | 2 | 2.6 | 1 | 33.3 |
|  |  |  |  |  |  |  |  |  |  | **(1.13-5.96)** |  |  |  |  |  |  |  |  |  |  |  |
| h8 | *GGACAC* | 0 | 0 |  |  | 1 | 0.6 | 0 | 0 |  |  | 0 | 0 | 0 | 0 |  |  | 1 | 1.3 | 0 | 0 |

*FCN2* - ficolin 2. N = number of chromosomes

LE – Leprosy patients, LL – Lepromatous leprosy, NL – Non-lepromatous leprosy.

HBV+ - with past or present hepatitis B infection, as judged by positive anti-HBc or HBsAg sorological results, respectively.

OR – odds ratio, CI – confidence interval, p – two-tailed p value, h – haplotype.

In bold: significant difference for haplotype frequencies, obtained with the exact Fisher’s test (only results with p values < 0.1 are given. All comparisons done with controls were made with leprosy HBV+ patients, unless otherwise stated).

& Association with *GGG___* haplotypes (*GGGCAC* and *GGGAAC*)

$ comparing Controls and HBV- leprosy patients: OR=0.18 (95%CI= 0.04-0.77), p=0.008

The following polymorphisms compose *FCN2* promoter – exon 1 haplotypes (in order of appearance in the [NC_000009](https://www.ensembl.org/Homo_sapiens/Location/View?contigviewbottom=variation_feature_variation%3Dnormal;db=core;source=dbSNP;v=rs3124952;vdb=variation;vf=686421819).12 reference sequence, preceded by their common name and with the corresponding nucleotides, within parentheses): *-986* variant: *g.134879836A>G,* rs3124952 (*A/G*); *-602* variant: *g.134880220A>G,* rs3124953 (*A/G*); *-557* variant: *g.134880265A>G,* rs3811140 (*A/G*); *-64* variant: *g.134880758A>C,* rs7865453 (*A/C*); *-4* variant: *g.134880818A>G,* rs17514136 (*A/G*); *+33* variant: *g.134880854C>T,* p.Gly11=, rs55797213 (*C/T*).

# no published nomenclature yet.
