# Supplementary material for: Hepatitis B Virus Infection Among Leprosy Patients: A Case for Polymorphisms Compromising Activation of the Lectin Pathway and Complement Receptors
Source: Front Immunol. 2021 Feb 11;11:574457. doi: 10.3389/fimmu.2020.574457 (PMC7904891; doi:10.3389/fimmu.2020.574457)
Supplement: Supplementary file 6 [file Table_5.docx]

Supplementary Material

# Supplementary Table 5. Distribution of *FCN2* exon 8 haplotypes in leprosy patients, according to HBV infection and severity of leprosy disease (lepromatous or not).

| *FCN2* | Exon 8 | Co |  | LE |  | LE |  | LL |  | LL |  | OR | p | NL |  | NL |  | OR | p |
| --- | --- | --- | --- | --- | --- | --- | --- | --- | --- | --- | --- | --- | --- | --- | --- | --- | --- | --- | --- |
| Haplotype # | Sequence | HBV- |  | HBV- |  | HBV+ |  | HBV- |  | HBV+ |  | (95%CI) |  | HBV- |  | HBV+ |  | (95%CI) |  |
| N |  | 268 | % | 150 | % | 102 | % | 80 | % | 76 | % |  |  | 70 | % | 26 | % |  |  |
| h1 | *MAG* | 64 | 23.88 | 44 | 29.33 | 23 | 22.55 | 29 | 36.25 | 13 | 17.11 | **0.36** | **0.011** | 15 | 21.43 | 10 | 38.46 |  |  |
|  |  |  |  |  |  |  |  |  |  |  |  | **(0.17-0.77)** |  |  |  |  |  |  |  |
| h2 | *MAT* | 4 | 1.49 | 0 | 0 | 0 | 0 | 0 | 0 | 0 | 0 |  |  | 0 | 0 | 0 | 0 |  |  |
| h3 | *TAG* | 12 | 4.48 | 4 | 2.67 | 5 | 4.90 | 2 | 2.5 | 5 | 6.58 | **2.19 &** | **0.022** | 2 | 2.86 | 0 | 0 | **0.32 &** | **0.028** |
|  |  |  |  |  |  |  |  |  |  |  |  | **(1.14-4.23)** |  |  |  |  |  | **(0.13-0.81)** |  |
| h4 | *TAT* | 159 | 59.33 | 88 | 58.67 | 60 | 58.82 | 39 | 48.75 | 48 | 63.16 | 1.80 | 0.078 | 49 | 70 | 12 | 46.15 | 0.37 | 0.055 |
|  |  |  |  |  |  |  |  |  |  |  |  | (0.95-3.42) |  |  |  |  |  | (0.15-0.93) |  |
| h5 | *TSG* | 29 | 10.82 | 14 | 9.33 | 14 | 13.73 | 10 | 12.5 | 10 | 13.16 |  |  | 4 | 5.71 | 4 | 15.38 |  |  |

*FCN2* - ficolin 2. N = number of chromosomes

LE – Leprosy patients, LL – Lepromatous leprosy, NL – Non-lepromatous leprosy.

HBV+ - with past or present hepatitis B infection, as judged by positive anti-HBc or HBsAg sorological results, respectively.

OR – odds ratio, CI – confidence interval, p – two-tailed p value, h – haplotype. *#* no nomenclature published yet.

In bold: significant difference for haplotype frequencies, obtained with the exact Fisher’s test (only results with p values < 0.1 are given).

Underlined: aminoacid one-letter symbols (shown in the haplotype sequence, in the case of missense mutations)

& Association with *TA* haplotypes (encoding threonine at position 236 and alanine at position 258 of the ficolin-2 protein)

The following polymorphisms compose *FCN2* promoter – exon 8 haplotypes (in order of appearance in the [NC_000009](https://www.ensembl.org/Homo_sapiens/Location/View?contigviewbottom=variation_feature_variation%3Dnormal;db=core;source=dbSNP;v=rs3124952;vdb=variation;vf=686421819).12 reference sequence, preceded by their common name and with the corresponding nucleotides, within parentheses): *+6359* variant: *g.134887180C>T,* p.Thr236Met, rs17549193 (*C/T*); *+6424* variant: *g.134887245G>T*, p.Ala258Ser, rs7851696 (*G/T*); *+6639* variant: *g.134887460T>G,* rs4521835 (*T/G*)*.*

# no published nomenclature yet.
