# Supplementary material for: Hepatitis B Virus Infection Among Leprosy Patients: A Case for Polymorphisms Compromising Activation of the Lectin Pathway and Complement Receptors
Source: Front Immunol. 2021 Feb 11;11:574457. doi: 10.3389/fimmu.2020.574457 (PMC7904891; doi:10.3389/fimmu.2020.574457)
Supplement: Supplementary file 7 [file Table_6.docx]

Supplementary Material

# Supplementary Table 6. Distribution of *FCN3* haplotypes in leprosy patients, according to HBV infection and severity of leprosy disease (lepromatous or not).

| *FCN3* | Exon 5 – intron 7 | Co |  | LE |  | LE |  | OR | p | LL |  | LL |  | NL |  | NL |  | OR | p |
| --- | --- | --- | --- | --- | --- | --- | --- | --- | --- | --- | --- | --- | --- | --- | --- | --- | --- | --- | --- |
| Haplotype # | Sequence | HBV- |  | HBV- |  | HBV+ |  | (95%CI) |  | HBV- |  | HBV+ |  | HBV- |  | HBV+ |  | (95%CI) |  |
| N |  | 292 | % | 184 | % | 102 | % |  |  | 102 | % | 72 | % | 62 | % | 16 | % |  |  |
| **1* | *C ins C* | 8 | 2.74 | 7 | 3.80 | 3 | 2.94 |  |  | 3 | 2.94 | 1 | 1.39 | 3 | 4.84 | 2 | 12.5 |  |  |
| **2A* | *C del C* | 209 | 71.58 | 133 | 72.28 | 70 | 68.63 |  |  | 69 | 67.65 | 51 | 70.83 | 49 | 79.03 | 9 | 56.25 |  |  |
| **2B1* | *C ins A* | 71 | 24.32 | 43 | 23.37 | 25 | 24.51 |  |  | 29 | 28.43 | 18 | 25 | 10 | 16.13 | 3 | 18.75 |  |  |
| **2B2* | *Del ins C* | 2 | 0.68 | 0 | 0 | 0 | 0 |  |  | 0 | 0 | 0 | 0 | 0 | 0 | 0 | 0 |  |  |
| ****2B2.2A*** | ***Del del C*** | 2 | 0.68 | 1 | 0.54 | 4 | 3.92 | 7.47 | 0.056 | 1 | 0.98 | 2 | 2.78 | 0 | 0 | 2 | 12.50 | --- | **0.04** |
|  |  |  |  |  |  |  |  | (0.82-67.75) |  |  |  |  |  |  |  |  |  |  |  |

*FCN3* - ficolin 3. N = number of chromosomes

LE – Leprosy patients. LL – Lepromatous leprosy. NL – Non-lepromatous leprosy.

HBV+ - with past or present hepatitis B infection, as judged by positive anti-HBc or HBsAg sorological results, respectively.

OR – odds ratio. CI – confidence interval. p – two-tailed p value.

In bold: significant difference for haplotype frequencies. obtained with the exact Fisher’s test (only results with p values < 0.1 are given).

The following polymorphisms compose *FCN3* exon 5 – intron 7 haplotypes (in order of appearance in the [NC_000001](https://www.ensembl.org/Homo_sapiens/Location/View?contigviewbottom=variation_feature_variation%20%200normal;db=core;source=dbSNP;v=rs532781899;vdb=variation;vf=21608417).11 reference sequence. preceded by their common name and with the corresponding nucleotides. within parentheses): *+1637* variant: *g.27373182del*, rs532781899 (*delC -* causing a frameshift and a truncated protein); *+3524_3532* variant: *g.27371297_27371298insTATTTGGCC,* rs28362807 (*insGGCCAAATA*) and *+4473* variant: *g.27370346G>T.* rs4494157 (*C/A*)*.*

# Phylogenetic nomenclature published by (23).
