# Supplementary material for: Hepatitis B Virus Infection Among Leprosy Patients: A Case for Polymorphisms Compromising Activation of the Lectin Pathway and Complement Receptors
Source: Front Immunol. 2021 Feb 11;11:574457. doi: 10.3389/fimmu.2020.574457 (PMC7904891; doi:10.3389/fimmu.2020.574457)
Supplement: Supplementary file 8 [file Table_7.docx]

Supplementary Material

# Supplementary Table 7. Distribution of *MASP1* haplotypes in leprosy patients. according to HBV infection and severity of leprosy disease (lepromatous or not).

| *MASP1* | Intron 1 - exon 12 | Co |  | OR | p | LE |  | LE |  | OR | p | LL |  | LL |  | OR | p | NL |  | NL |  |
| --- | --- | --- | --- | --- | --- | --- | --- | --- | --- | --- | --- | --- | --- | --- | --- | --- | --- | --- | --- | --- | --- |
| Haplotype # | Sequence | HBV- |  | (95%CI) |  | HBV- |  | HBV+ |  | (95%CI) |  | HBV- |  | HBV+ |  | (95%CI) |  | HBV- |  | HBV+ |  |
| N |  | 392 | % |  |  | 216 | % | 144 | % |  |  | 122 | % | 104 | % |  |  | 70 | % | 24 | % |
| **h1** | ***AC_CCA*** | 1 | 0.26 | **11.17** | **0.02** | 1 | 0.46 | 4 | 2.78 | **2.5 &** | **0.009** | 1 | 0.82 | 2 | 1.92 |  |  | 0 | 0 | 1 | 4.17 |
|  |  |  |  | **(1.24-100.81)** |  |  |  |  |  | **(1.28-4.89)** |  |  |  |  |  |  |  |  |  |  |  |
| **h2** | ***AC_CCG*** | 39 | 9.95 |  |  | 15 | 6.94 | 20 | 13.89 | **2.16** | **0.044** | 5 | 4.10 | 15 | 14.42 | **3.94** | **0.009** | 5 | 7.14 | 5 | 20.83 |
|  |  |  |  |  |  |  |  |  |  | **(1.07-4.38)** |  |  |  |  |  | **(1.38-11.26)** |  |  |  |  |  |
| **h3** | ***AC_CTG*** | 10 | 2.55 |  |  | 9 | 4.17 | 1 | 0.69 | 0.16 | 0.055 | 6 | 4.92 | 0 | 0 | --- | **0.03** | 1 | 1.43 | 0 | 0 |
|  |  |  |  |  |  |  |  |  |  | (0.02-1.28) |  |  |  |  |  |  |  |  |  |  |  |
| h4 | *AC_GTG* | 4 | 1.02 |  |  | 0 | 0 | 0 | 0 |  |  | 0 | 0 | 0 | 0 |  |  | 0 | 0 | 0 | 0 |
| **h5** | ***GC_CCA*** | 57 | 14.54 | **0.50 $** | **0.019** | 17 | 7.87 | 16 | 11.11 |  |  | 6 | 4.92 | 10 | 9.62 |  |  | 7 | 10 | 3 | 12.5 |
|  |  |  |  | **(0.28-0.89)** |  |  |  |  |  |  |  |  |  |  |  |  |  |  |  |  |  |
| **h6** | ***GC_CCG*** | 82 | 20.92 | **1.81 $** | **0.002** | 70 | 32.41 | 36 | 25.0 |  |  | 39 | 31.97 | 26 | 25 |  |  | 25 | 35.71 | 6 | 25 |
|  |  |  |  | **(1.25-2.64)** |  |  |  |  |  |  |  |  |  |  |  |  |  |  |  |  |  |
| h7 | *GC_CTG* | 56 | 14.29 |  |  | 30 | 13.89 | 17 | 11.81 |  |  | 16 | 13.11 | 13 | 12.5 |  |  | 10 | 14.29 | 2 | 8.33 |
| h8 | *GC_GTG* | 36 | 9.18 |  |  | 12 | 5.56 | 10 | 6.94 |  |  | 8 | 6.56 | 9 | 8.65 |  |  | 7 | 10 | 1 | 4.17 |
| h9 | *GT_CCA* | 19 | 4.85 |  |  | 10 | 4.63 | 5 | 3.47 |  |  | 8 | 6.56 | 4 | 3.85 |  |  | 0 | 0 | 1 | 4.17 |
| h10 | *GT_CCG* | 62 | 15.82 |  |  | 25 | 11.57 | 21 | 14.58 |  |  | 16 | 13.11 | 13 | 12.5 |  |  | 8 | 11.43 | 4 | 16.67 |
| h11 | *GT_CTG* | 23 | 5.87 |  |  | 21 | 9.72 | 13 | 9.03 |  |  | 14 | 11.48 | 11 | 10.58 |  |  | 7 | 10 | 1 | 4.17 |
| h12 | *GT_GTG* | 3 | 0.77 |  |  | 6 | 2.78 | 1 | 0.69 |  |  | 3 | 2.46 | 1 | 0.96 |  |  | 0 | 0 | 0 | 0 |

*MASP1* – Mannan-binding lectin-associated serine protease 1. N = number of chromosomes

LE – Leprosy patients, LL – Lepromatous leprosy, NL – Non-lepromatous leprosy.

HBV+ - with past or present hepatitis B infection, as judged by positive anti-HBc or HBsAg serological results, respectively.

OR – odds ratio, CI – confidence interval, p – two-tailed p value, h – haplotype.

In bold: significant difference for haplotype frequencies. obtained with the exact Fisher’s test (only results with p values < 0.1 are given. All comparisons done with controls were made with leprosy HBV+ patients, unless otherwise stated).

& Association with *AC_CC* haplotypes (*AC_CCA* and *AC_CCG*)

$ comparing Controls and HBV- leprosy patients

The following polymorphisms compose *MASP1* intron 1 – exon 12 haplotypes (in order of appearance in the [NC_000003](https://www.ensembl.org/Homo_sapiens/Location/View?contigviewbottom=variation_feature_variation%20%200normal;db=core;source=dbSNP;v=rs7609662;vdb=variation;vf=2441649).12 reference sequence and with the corresponding nucleotides. within parentheses): *g.187288910C>T* variant: rs7609662 (*G/A*); *g.187288228G>A* variant: rs13064994 (*C/T*); *g.187235126G>C* variant: rs72549262 (*C/G*); *g.187234800G>A* variant: rs1109452 (*C/T*) and *g.187234799C>T* variant: rs850314 (*G/A*).

*#* no nomenclature published yet.
