# Supplementary material for: Hepatitis B Virus Infection Among Leprosy Patients: A Case for Polymorphisms Compromising Activation of the Lectin Pathway and Complement Receptors
Source: Front Immunol. 2021 Feb 11;11:574457. doi: 10.3389/fimmu.2020.574457 (PMC7904891; doi:10.3389/fimmu.2020.574457)
Supplement: Supplementary file 9 [file Table_8.docx]

Supplementary Material

# Supplementary Table 8. Distribution of *MASP2* haplotypes in leprosy patients. according to HBV infection and severity of leprosy disease (lepromatous or not).

| *MASP2* | Promoter - exon 12 | Co |  | OR | p | LE |  | LE |  | OR | p | LL |  | LL |  | OR | p | NL |  | NL |  |
| --- | --- | --- | --- | --- | --- | --- | --- | --- | --- | --- | --- | --- | --- | --- | --- | --- | --- | --- | --- | --- | --- |
| Haplotype # | Sequence | HBV- |  | (95%CI) |  | HBV- |  | HBV+ |  | (95%CI) |  | HBV- |  | HBV+ |  | (95%CI) |  | HBV- |  | HBV+ |  |
| N |  | 816 | % |  |  | 224 | % | 146 | % |  |  | 124 | % | 106 | % |  |  | 74 | % | 40 | % |
| **1A [AG]* | *CRDPAGCDVRC* | 30 | 3.68 |  |  | 11 | 4.91 | 6 | 4.11 |  |  | 7 | 5.65 | 4 | 3.77 |  |  | 2 | 2.7 | 2 | 5 |
| **1A [GA]* | *CRDPGACDVRC* | 11 | 1.35 |  |  | 4 | 1.79 | 0 | 0 |  |  | 2 | 1.61 | 1 | 0.94 |  |  | 1 | 1.35 | 0 | 0 |
| **1B1-h [AG]* | *CRDPAGTDVRC* | 9 | 1.10 |  |  | 5 | 2.23 | 3 | 2.05 |  |  | 3 | 2.42 | 1 | 0.94 |  |  | 2 | 2.7 | 2 | 5 |
| **1B1-h [GA]* | *CRDPGATDVRC* | 105 | 12.87 |  |  | 31 | 13.8 | 12 | 8.22 |  |  | 19 | 15.3 | 10 | 9.43 |  |  | 8 | 10.8 | 2 | 5 |
| **1B2-h* | *CQDPGATDVRC* | 9 | 1.10 |  |  | 5 | 2.23 | 4 | 2.74 |  |  | 2 | 1.61 | 1 | 0.94 |  |  | 2 | 2.7 | 3 | 7.5 |
| **1C1-l* | *CRDLAGCDVRC* | 4 | 0.49 |  |  | 3 | 1.34 | 2 | 1.37 |  |  | 2 | 1.61 | 1 | 0.94 |  |  | 1 | 1.35 | 1 | 2.5 |
| **1C2-l* | ***CRDLAGCDVHC*** | 6 | 0.74 | **6.8** | **0.001** | 3 | 1.34 | 7 | 4.79 | 3.71 | 0.055 | 1 | 0.81 | 5 | 4.72 | 6.09 | 0.097 | 2 | 2.7 | 2 | 5 |
|  |  |  |  | **2.25-20.53** |  |  |  |  |  | 0.94-14.59 |  |  |  |  |  | 0.7-52.97 |  |  |  |  |  |
| **2A1* | *CRDPAGCDVRT* | 3 | 0.37 |  |  | 0 | 0 | 2 | 1.37 |  |  | 0 | 0 | 2 | 1.89 |  |  | 0 | 0 | 0 | 0 |
| **2A2-l* | *CRDPAGCDART* | 37 | 4.53 |  |  | 6 | 2.68 | 5 | 3.42 |  |  | 4 | 3.23 | 4 | 3.77 |  |  | 2 | 2.7 | 1 | 2.5 |
| **2B2A-i [AG]* | *ARDPAGCYVRT* | 538 | 65.93 |  |  | 145 | 64.7 | 91 | 62.3 |  |  | 77 | 62.1 | 71 | 67 |  |  | 51 | 68.9 | 20 | 50 |
| **2B2A-i [GG]* | *ARDPGGCYVRT* | 0 | 0 |  |  | 0 | 0 | 1 | 0.69 |  |  | 0 | 0 | 1 | 0.94 |  |  | 0 | 0 | 0 | 0 |
| **2B2A-i [GA]* | *ARDPGACYVRT* | 6 | 0.74 |  |  | 0 | 0 | 0 | 0 |  |  | 0 | 0 | 0 | 0 |  |  |  |  |  |  |
| **2B1-i [AG]* | ***CRDPAGCYVRT*** | 33 | 4.04 | **0.21 $** | **0.02** | 2 | 0.89 | 4 | 2.74 | **4.48 !** | **0.012** | 1 | 0.81 | 1 | 0.94 |  |  | 1 | 1.35 | 3 | 7.5 |
|  |  |  |  | **0.05-0.90** |  |  |  |  |  | **1.40-14.36** |  |  |  |  |  |  |  |  |  |  |  |
| **2B1-i [GA]* | ***CRDPGACYVRT*** | 15 | 1.84 | **0.29 !$** | **0.009** | 2 | 0.89 | 7 | 4.79 | **5.59** | **0.032** | 1 | 0.81 | 4 | 3.77 |  |  | 1 | 1.35 | 2 | 5 |
|  |  |  |  | **0.10-0.82** |  |  |  |  |  | **1.14-27.29** |  |  |  |  |  |  |  |  |  |  |  |
| **2B2B-l* | ***ARGPAGCYVRT*** | 8 | 0.98 | **3.26 $** | **0.026** | 7 | 3.12 | 2 | 1.37 |  |  | 5 | 4.03 | 0 | 0 | --- | 0.064 | 1 | 1.35 | 2 | 5 |
|  |  |  |  | **1.17-9.08** |  |  |  |  |  |  |  |  |  |  |  |  |  |  |  |  |  |
| **2B2A.1B1a [AG]* | *ARDPAGTDVRC* | 1 | 0.12 |  |  | 0 | 0 | 0 | 0 |  |  | 0 | 0 | 0 | 0 |  |  | 0 | 0 | 0 | 0 |
| **2B2A.1B1b [AG]* | *ARDPAGCYVRC* | 1 | 0.12 |  |  | 0 | 0 | 0 | 0 |  |  | 0 | 0 | 0 | 0 |  |  | 0 | 0 | 0 | 0 |

*MASP2* – Mannan-binding lectin-associated serine protease 2. N = number of chromosomes.

LE – Leprosy patients. LL – Lepromatous leprosy. NL – Non-lepromatous leprosy.

HBV+ - with past or present hepatitis B infection, as judged by positive anti-HBc or HBsAg serological results, respectively.

OR – odds ratio, CI – confidence interval, p – two-tailed p value.

In bold: significant difference for haplotype frequencies. obtained with the exact Fisher’s test (only results with p values < 0.1 are given. all comparisons done with controls were made with leprosy HBV+ patients. unless otherwise stated).

Underlined: aminoacid one-letter symbols (shown in the haplotype sequence, in the case of missense mutations)

$ comparing Controls and HBV- leprosy patients

! Association with *CRDP_CYVRT* haplotypes (**2B1-i*)

The following polymorphisms compose *MASP2* promoter – exon 12 haplotypes (in order of appearance in the [NC_000001](https://www.ensembl.org/Homo_sapiens/Location/View?contigviewbottom=variation_feature_variation%3Dnormal;db=core;source=dbSNP;v=rs7548659;vdb=variation;vf=3505637).11 reference sequence and with the corresponding nucleotides. within parentheses): *g.11047382G>T* variant: rs7548659 (*C/A*); *g.11046672C>T* variant: p.Arg99Gln, rs61735600 (*G/A*); *g.11046609T>C* variant: p.Asp120Gly, rs72550870 (*A/G*); *g.11046591G>A* variant: p.Pro126Leu, rs56392418 (*C/T*); *g.11045065C>T* variant: rs2273344 (*G/A*); *g.11044788T>C* variant: rs9430347 (*A/G*); *g.11031148G>A* variant: rs17409276 (*C/T*); *g.11030859C>A* variant: p.Asp371Tyr, rs12711521 (*G/T*); *g.11030840A>G* variant: p.Val377Ala, rs2273346 (*T/C*); *g.11027630C>T* variant: p.Arg439His, rs12085877 (*G/A*); *g.11027467G>A* variant: rs1782455 (*C/T*).

*#* Phylogenetic nomenclature published by (61), updated by (20).
