# Supplementary material for: Hepatitis B Virus Infection Among Leprosy Patients: A Case for Polymorphisms Compromising Activation of the Lectin Pathway and Complement Receptors
Source: Front Immunol. 2021 Feb 11;11:574457. doi: 10.3389/fimmu.2020.574457 (PMC7904891; doi:10.3389/fimmu.2020.574457)
Supplement: Supplementary file 10 [file Table_9.docx]

Supplementary Material

# Supplementary Table 9. Distribution of *CR1* haplotypes in leprosy patients. according to HBV infection and severity of leprosy disease (lepromatous or not).

| *CR1* | Intron 4 – intron 37 | Co |  | OR | p | LE |  | LE |  | OR | p | LL |  | LL |  | NL |  | NL |  | OR | p |
| --- | --- | --- | --- | --- | --- | --- | --- | --- | --- | --- | --- | --- | --- | --- | --- | --- | --- | --- | --- | --- | --- |
| Haplotype # | Sequence | HBV- |  | (95%CI) |  | HBV- |  | HBV+ |  | (95%CI) |  | HBV- |  | HBV+ |  | HBV- |  | HBV+ |  | (95%CI) |  |
| N |  | 396 | % |  |  | 192 | % | 112 | % |  |  | 110 | % | 74 | % | 58 | % | 22 | % |  |  |
| **1* | *GCHTCTLCG* | 36 | 9.09 |  |  | 27 | 14.06 | 15 | 13.39 |  |  | 19 | 17.27 | 12 | 16.22 | 5 | 8.62 | 2 | 9.09 |  |  |
| **2* | *GCHTCTECG* | 9 | 2.27 |  |  | 4 | 2.08 | 2 | 1.79 |  |  | 3 | 2.73 | 1 | 1.35 | 0 | 0 | 1 | 4.55 |  |  |
| ****3A1*** | ***GCRTCTLCG*** | 11 | 2.78 | 2.33 | 0.087 | 2 | 1.04 | 7 | 6.25 | **6.33** | **0.014** | 1 | 0.91 | 2 | 2.70 | 1 | 1.72 | 5 | 22.73 |  |  |
|  |  |  |  | (0.88-6.16) |  |  |  |  |  | **(1.29-31.04)** |  |  |  |  |  |  |  |  |  |  |  |
| ****3A2A*** | ***GCRTTTLCG*** | 34 | 8.59 |  |  | 11 | 5.73 | 6 | 5.36 | **2.23 &** | **0.012** | 7 | 6.36 | 3 | 4.05 | 3 | 5.17 | 2 | 9.09 | **7.34 &** | **0.002** |
|  |  |  |  |  |  |  |  |  |  | **(1.22-4.06)** |  |  |  |  |  |  |  |  |  | **(2.1-25.62)** |  |
| ****3A2B*** | ***GCRTTCLCG*** | 40 | 10.1 |  |  | 12 | 6.25 | 15 | 13.39 | **2.32** | **0.039** | 9 | 8.18 | 11 | 14.86 | 1 | 1.72 | 2 | 9.09 |  |  |
|  |  |  |  |  |  |  |  |  |  | **(1.04-5.15)** |  |  |  |  |  |  |  |  |  |  |  |
| **3B1* | *GCHTCTLCA* | 78 | 19.70 |  |  | 35 | 18.23 | 18 | 16.07 |  |  | 20 | 18.18 | 13 | 17.57 | 10 | 17.24 | 4 | 18.18 |  |  |
| **3B2A* | *GTHTCTLCA* | 12 | 3.03 |  |  | 6 | 3.13 | 2 | 1.79 |  |  | 4 | 3.64 | 2 | 2.70 | 2 | 3.45 | 0 | 0 |  |  |
| **3B2B* | *GTHMCTLCA* | 68 | 17.17 |  |  | 33 | 17.19 | 19 | 16.96 |  |  | 13 | 11.82 | 12 | 16.22 | 14 | 24.14 | 4 | 18.18 |  |  |
| **4* | *ACHTCTLAG* | 61 | 15.40 |  |  | 34 | 17.71 | 17 | 15.18 |  |  | 17 | 15.45 | 11 | 14.86 | 14 | 24.14 | 1 | 4.55 | 0.15 | 0.056 |
|  |  |  |  |  |  |  |  |  |  |  |  |  |  |  |  |  |  |  |  | (0.02-1.22) |  |
| **1.3B2B* | *GTHMCTLCA* | 21 | 5.30 |  |  | 7 | 3.65 | 2 | 1.79 |  |  | 4 | 3.64 | 1 | 1.35 | 3 | 5.17 | 0 | 0 |  |  |
| **3B2B.1* | *GTHMCTLCG* | 2 | 0.51 |  |  | 4 | 2.08 | 2 | 1.79 |  |  | 2 | 1.82 | 1 | 1.35 | 1 | 1.72 | 0 | 0 |  |  |
| **3B2B.3A2B.3B1* | *GTHMCCLCA* | 9 | 2.27 |  |  | 10 | 5.21 | 6 | 5.36 |  |  | 6 | 5.45 | 4 | 5.41 | 2 | 3.45 | 1 | 4.55 |  |  |
| **1.3A2B.3B2B* | *GCHTCCLCA* | 9 | 2.27 |  |  | 4 | 2.08 | 1 | 0.89 |  |  | 3 | 2.73 | 1 | 1.35 | 1 | 1.72 | 0 | 0 |  |  |
| **1.3B2B.1* | *GCHMCTLCG* | 6 | 1.52 |  |  | 2 | 1.04 | 0 | 0 |  |  | 1 | 0.91 | 0 | 0 | 1 | 1.72 | 0 | 0 |  |  |
| **1.3B2B.3A2B.3B1* | *GCHTCCLCA* | 0 | 0 |  |  | 1 | 0.52 | 0 | 0 |  |  | 1 | 0.91 | 0 | 0 | 0 | 0 | 0 | 0 |  |  |

*CR1* – complement receptor 1. N = number of chromosomes

Co – controls, LE – Leprosy patients, LL – Lepromatous leprosy, NL – Non-lepromatous leprosy.

HBV+ - with past or present hepatitis B infection, as judged by positive anti-HBc or HBsAg sorological results, respectively.

OR – odds ratio, CI – confidence interval, p – two-tailed p value.

In bold: significant difference for haplotype frequencies. obtained with the exact Fisher’s test (only results with p values < 0.1 are given. All comparisons done with controls were made with leprosy HBV+ patients).

Underlined: aminoacid one-letter symbols (shown in the haplotype sequence, in the case of missense mutations)

& Association with all **3A* haplotypes (encoding arginine at the 1208 position of the CR1 protein)

The following polymorphisms compose *CR1* intron 4 – intron 37 haplotypes (in order of appearance in the [NC_000001](https://www.ensembl.org/Homo_sapiens/Location/View?contigviewbottom=variation_feature_variation%3Dnormal;db=core;source=dbSNP;v=rs7548659;vdb=variation;vf=3505637).11 reference sequence and with the corresponding nucleotides within parentheses): *g.207518704A>G* variant: rs6656401 (*A/*G); *g.207579645C>T* variant: rs3849266 (*C/T*); *g.207580276A>G* variant: p.His1208Arg, rs2274567 (*A/G*); *g.207587428C>T* variant: p.Thr1408Met, rs3737002 (*C/T*); *g.207587851C>T* variant: rs11118131 (*C/T*); *g.207608809T>C* variant: rs11118167 (*T/C*); *g.207609511A>G* variant: p.Lys1590Glu, rs17047660 (*A/G*); *g.207629207A>C* variant: rs4844610 (*A/C*); *g.207630250G>A* variant: rs12034383 (*G/A*).

*#* Phylogenetic nomenclature published by (22).
