# Supplementary material for: Hepatitis B Virus Infection Among Leprosy Patients: A Case for Polymorphisms Compromising Activation of the Lectin Pathway and Complement Receptors
Source: Front Immunol. 2021 Feb 11;11:574457. doi: 10.3389/fimmu.2020.574457 (PMC7904891; doi:10.3389/fimmu.2020.574457)
Supplement: Supplementary file 11 [file Table_10.docx]

Supplementary Material

# Supplementary Table 10. Distribution of *VSIG4* haplotypes in leprosy patients. according to HBV infection and severity of leprosy disease (lepromatous or not).

| *VS1G4* | Promoter – intron 3 | Co |  | OR | p | LE |  | LE |  | OR | p | LL |  | LL |  | OR | p | NL |  | NL |  |
| --- | --- | --- | --- | --- | --- | --- | --- | --- | --- | --- | --- | --- | --- | --- | --- | --- | --- | --- | --- | --- | --- |
| Haplotype # | Sequence | HBV- |  | (95%CI) |  | HBV- |  | HBV+ |  | (95%CI) |  | HBV- |  | HBV+ |  | (95%CI) |  | HBV- |  | HBV+ |  |
| N |  | 242 | % |  |  | 157 | % | 99 | % |  |  | 82 | % | 69 | % |  |  | 59 | % | 19 | % |
| h1 | ***TCGRCG*** | 36 | 14.88 | **2.11 &** | **0.009** | 24 | 15.29 | 21 | 21.21 | **1.93 &** | **0.034** | 14 | 17.07 | 15 | 21.74 | 2.09 & | 0.060 | 7 | 11.86 | 3 | 15.79 |
|  |  |  |  | **(1.23-3.61)** |  |  |  |  |  | **(1.08-3.45)** |  |  |  |  |  | (0.98-4.45) |  |  |  |  |  |
| h2 | *TCGWCG* | 1 | 0.41 |  |  | 1 | 0.64 | 1 | 1.01 |  |  | 1 | 1.22 | 1 | 1.45 |  |  | 0 | 0 | 0 | 0 |
| h3 | ***TGARTA*** | 191 | 78.93 | **0.56** | **0.037** | 125 | 79.62 | 67 | 67.68 | **0.54** | **0.038** | 65 | 79.27 | 46 | 66.67 | 0.52 | 0.097 | 48 | 81.36 | 13 | 68.42 |
|  |  |  |  | **(0.33-0.94)** |  |  |  |  |  | **(0.30-0.91)** |  |  |  |  |  | (0.25-1.09) |  |  |  |  |  |
| h4 | ***TGGRCG*** | 7 | 2.89 | **3.77** | **0.011** | 6 | 3.82 | 10 | 10.10 | 2.83 | 0.062 | 1 | 1.22 | 7 | 10.14 | **9.15** | **0.024** | 4 | 6.78 | 3 | 15.79 |
|  |  |  |  | **(1.39-10.21)** |  |  |  |  |  | (0.99-8.0) |  |  |  |  |  | **(1.09-76.29)** |  |  |  |  |  |
| h5 | *TGGRTA* | 0 | 0 |  |  | 1 | 0.64 | 0 | 0 |  |  | 1 | 1.22 | 0 | 0 |  |  | 0 | 0 | 0 | 0 |
| h6 | *TCARTA* | 5 | 2.07 |  |  | 0 | 0 | 0 | 0 |  |  | 0 | 0 | 0 | 0 |  |  | 0 | 0 | 0 | 0 |
| h7 | *TGARCG* | 2 | 0.82 |  |  | 0 | 0 | 0 | 0 |  |  | 0 | 0 | 0 | 0 |  |  | 0 | 0 | 0 | 0 |

*VSIG4* - V-set and immunoglobulin domain containing 4. N = number of chromosomes

Co – controls, LE – Leprosy patients, LL – Lepromatous leprosy, NL – Non-lepromatous leprosy.

HBV+ - with past or present hepatitis B infection, as judged by positive anti-HBc or HBsAg serological results, respectively.

OR – odds ratio, CI – confidence interval, p – two-tailed p value, h – haplotype.

In bold: significant difference for haplotype frequencies. obtained with the exact Fisher’s test (only results with p values < 0.1 are given. . all comparisons done with controls were made with leprosy HBV+ patients).

Underlined: aminoacid one-letter symbols (shown in the haplotype sequence, in the case of missense mutations).

& Association with *GRCG* haplotypes (*TCGRCG* and *TGGRCG*)

The following polymorphisms compose *VSIG4* promoter – intron 3 haplotypes (in order of appearance in the [NC_000023](https://www.ensembl.org/Homo_sapiens/Location/View?contigviewbottom=variation_feature_variation%3Dnormal;db=core;source=dbSNP;v=rs2284705;vdb=variation;vf=141337374).11 reference sequence and with the corresponding nucleotides. within parentheses): *g.66041429A>T* variant: rs2284705 (*T/A*); *g.66040683C>G* variant: rs5964489 (*G/C*); *g.66033927T>C* variant: rs5964488 (*G/A*); *g.66033564G>A* variant: p.Arg108Trp, rs34581041 (*C/T*); *g.66029762A>G* variant: rs5964487 (*T/C*); *g.66029097T>C* variant: rs9887348 (*A/G*).

*#* no nomenclature published yet.
