# Supplementary material for: Hepatitis B Virus Infection Among Leprosy Patients: A Case for Polymorphisms Compromising Activation of the Lectin Pathway and Complement Receptors
Source: Front Immunol. 2021 Feb 11;11:574457. doi: 10.3389/fimmu.2020.574457 (PMC7904891; doi:10.3389/fimmu.2020.574457)
Supplement: Supplementary file 12 [file Table_11.docx]

Supplementary Material

# Supplementary Table 11. Distribution of *C3* haplotypes in leprosy patients. according to HBV infection and severity of leprosy disease (lepromatous or not).

| *C3* | Intron 2 – Exon 9 | Co |  | LE |  | LE |  | LL |  | LL |  | NL |  | NL |  |
| --- | --- | --- | --- | --- | --- | --- | --- | --- | --- | --- | --- | --- | --- | --- | --- |
| Haplotype # | Sequence | HBV- |  | HBV- |  | HBV+ |  | HBV- |  | HBV+ |  | HBV- |  | HBV+ |  |
| N |  | 382 | % | 186 | % | 120 | % | 102 | % | 82 | % | 66 | % | 24 | % |
| h1 | *ARP* | 231 | 60.47 | 119 | 63.98 | 76 | 63.33 | 66 | 64.71 | 53 | 64.63 | 41 | 62.12 | 14 | 58.33 |
| h2 | *AGP* | 9 | 2.36 | 3 | 1.61 | 1 | 0.83 | 3 | 2.94 | 1 | 1.22 | 0 | 0 | 0 | 0 |
| h3 | *AGL* | 40 | 10.47 | 18 | 9.68 | 12 | 10.00 | 11 | 10.78 | 9 | 10.98 | 5 | 7.58 | 2 | 8.33 |
| h4 | *GRP* | 101 | 26.44 | 46 | 24.73 | 31 | 25.83 | 22 | 21.57 | 19 | 23.17 | 20 | 30.3 | 8 | 33.33 |
| h5 | *GRL* | 1 | 0.26 | 0 | 0 | 0 | 0 | 0 | 0 | 0 | 0 | 0 | 0 | 0 | 0 |

C3 – complement component 3. N = number of chromosomes

Co – controls, LE – Leprosy patients, LL – Lepromatous leprosy, NL – Non-lepromatous leprosy, h – haplotype.

HBV+ - with past or present hepatitis B infection, as judged by positive anti-HBc or HBsAg serological results, respectively.

OR – odds ratio. CI – confidence interval. p – two-tailed p value.

In bold: significant difference for haplotype frequencies. obtained with the exact Fisher’s test (only results with p values < 0.1 are given. All comparisons done with controls were made with leprosy HBV+ patients).

Underlined: aminoacid one-letter symbols (shown in the haplotype sequence. in the case of missense mutations)

The following polymorphisms compose *C3* intron 2 – exon 9 haplotypes (in order of appearance in the NC_000019.10 reference sequence and with the corresponding nucleotides. within parentheses): *g.6718523T>C* variant: rs2250656 (*A/G*); *g.6718376G>C* variant: p.Arg102Gly, rs2230199 (*C/G*) and *g.6713251G>A* variant: p.Pro314Leu, rs1047286 (*C/T*).

*#* no nomenclature published yet.
